# tArp Is an Actin‐Related Protein Required for Ciliary Function That Is Conserved in Ciliates and Related Eukaryotic Groups

**DOI:** 10.1111/jeu.70123

**Published:** 2026-09-18

**Authors:** Miku Kagawa, Minori Hagita, Takuro Nakayama, Kogiku Shiba, Takako Kato‐Minoura, Shinya Matsuda, Kazuo Inaba, Yuji Inagaki, Kentaro Nakano

**Affiliations:** ^1^ Degree Programs in Biology, Graduate School of Science and Technology University of Tsukuba Tsukuba Ibaraki Japan; ^2^ Center for Computational Sciences University of Tsukuba Tsukuba Ibaraki Japan; ^3^ Shimoda Marine Research Center University of Tsukuba Shizuoka Japan; ^4^ Tateyama Marine Laboratory Institute for Marine and Coastal Research, Ochanomizu University Chiba Japan; ^5^ Department of Biological Sciences, School of Science and Engineering Chuo University Tokyo Japan

**Keywords:** actin‐related protein, cilia, SAR, tetrahymena

## Abstract

Cells exhibit a wide range of behaviors due to their diverse molecular systems, which have been established through the molecular evolution of individual species. Protein families generated through gene duplications may be involved in this process. The actin cytoskeleton is no exception. The majority of eukaryotes express not only actin, but also several types of actin‐related proteins (Arps). The ciliate *Tetrahymena thermophila* possesses a species‐specific Arp, named tArp, which localizes to cilia. However, the evolutionary origin of the gene and its detailed cellular functions remain unclear. In the present study, we performed a comprehensive molecular phylogenetic analysis of actin and Arps across eukaryotes, with a focus on ciliates and their close relatives, and demonstrated that tArp is widely conserved in SAR, one of the largest eukaryotic supergroups consisting of Stramenopiles, Alveolata, and Rhizaria. Furthermore, the gene disruption of *tARP* reduced swimming velocity due to defects in ciliary beat frequency and waveforms. Therefore, we propose that tArp is a member of the novel Arp family that specifically contributes to efficient ciliary motility.

## 1 Introduction

Actin forms flexible cytoskeletons and plays crucial roles in cell motility, cytokinesis, and the establishment of cell morphology (Pollard and Cooper 2009). It also functions as a component of cilia, although its role remains unclear, and as a component of the dynein‐dynactin complex, which is responsible for cytoplasmic transport. In addition to its functions in the cytoplasm, actin was recently shown to be involved in regulating gene expression in the nucleus (Kelpsch and Tootle 2018). Many eukaryotic cells express genes encoding actin‐related proteins (Arps), which likely evolved from actin or its ancestral gene through molecular evolution. The various cellular functions of actin mentioned above are not only conducted by canonical actin, but are also supported by Arps.

Arps are classified into several families, some of which are known to function in the cytosol. Arp1 and Arp11 (called Arp10 in budding yeast) function as subunits of the dynactin complex in cytoplasmic transport (Lau et al. 2021; Urnavicius et al. 2015; Clark and Rose 2006), while Arp2 and Arp3 form the complex that binds to the sides of actin filaments, thereby promoting the formation of an actin network at the leading edge required for cell movement (Goley and Welch 2006; Pollard and Borisy 2003). On the other hand, Arp4 ~9 function as components of chromatin remodeling complexes in the nucleus (Brahma et al. 2018; Kandasamy et al. 2009; Kitayama et al. 2009; Mizuguchi et al. 2004; Szerlong et al. 2003; Galarneau et al. 2000). Some of these Arps are conserved across multiple eukaryotic supergroups (Morozov et al. 2018; Meagher et al. 2009; Sehring, Mansfeld, et al. 2007; Gordon and Sibley 2005; Muller et al. 2005; Kandasamy et al. 2004), whereas other Arps are found only in specific groups of organisms. For example, Arp7 and Arp9 appear to be restricted to species belonging to the Ascomycota (Muller et al. 2005). In addition, animal‐specific Arps such as Arp‐T1 and Arp‐T2 have been reported (Sehring, Mansfeld, et al. 2007; Heid et al. 2002). Therefore, eukaryotic cells appear to utilize universal Arps and species‐specific Arps that appeared through molecular evolution at different stages of their evolutionary lineages.

The majority of eukaryotes possess cellular structures called cilia (also called flagella), which have a characteristic 9 + 2 arrangement of microtubules. This cellular structure is considered to have been acquired during the early stages of eukaryotic evolution and has been widely conserved throughout the long evolutionary process (Mitchell 2017; Satir 2017). The rhythmic beating of cilia is driven by axonemal dyneins, which generate sliding forces between adjacent outer‐doublet microtubules in the 9 + 2 axoneme (Ishikawa 2017). Axonemal dyneins are divided into outer‐arm and inner‐arm dyneins, the structures and functions of which differ. Structural biology and biochemical studies showed the presence of actin and its isoforms within cilia across diverse eukaryotic supergroups, including humans, *Ciona*, *Trypanosoma*, the green algae *Chlamydomonas*, and the ciliate *Tetrahymena* (Xia et al. 2025; Walton et al. 2023; Gui et al. 2021; Nakachi et al. 2011; Yanagisawa and Kamiya 2001; Muto et al. 1994). In *Tetrahymena*, biochemical fractionation revealed that actin was present in the fraction of the 14S inner arm dynein complex (Muto et al. 1994), which is considered to be the single‐head inner arm dynein (Marchese‐Ragona et al. 1988; Toyoshima 1987). Moreover, actin has been suggested to form complexes with single‐head inner arm dynein in conjunction with p28 or centrin in humans, *Trypanosoma*, and *Chlamydomonas* (Xia et al. 2025; Walton et al. 2023; Gui et al. 2021; Yanagisawa and Kamiya 2001). However, since actin is a protein that is essential for cellular function, the effects of its gene deficiency on ciliary movement remain unclear in most organisms. Previous studies reported that *Chlamydomonas* cells lacking normal actin function expressed the actin‐like protein NAP to compensate for the function of actin in the axoneme (Hirono et al. 2003; Kato‐Minoura et al. 1997, 1998). The knockout (KO) of the *ACT1* gene encoding a major actin isoform in *Tetrahymena thermophila* resulted in a severe abnormality in the ciliary motility as a terminal phenotype (Williams et al. 2006); however, the reason why actin plays such a crucial role in the ciliary structure remains unclear.

During the sequencing of the *T. thermophila* genome (Eisen et al. 2006), it was revealed that this organism possesses a unique Arp protein in its cilia named tArp (Kuribara et al. 2006), which is encoded by the *tARP* gene (TTHERM_00378580). It is referred to as ARP4 in the *Tetrahymena* Gene Database, but does not belong to the Arp4 family. No genes similar to *tARP* were found in the genomes of *Homo sapiens* , *Drosophila melanogaster* , *Saccharomyces cerevisiae* , or *Arabidopsis thaliana* , for which genome information was available at that time. To further investigate the phylogenetic distribution of tArp beyond the limited taxon sampling of the previous study, we herein performed a molecular phylogenetic analysis of tArp across a broad range of eukaryotes, focusing primarily on ciliates and their closely related groups. Furthermore, since tArp is localized to cilia and its expression increases during ciliary regeneration (Kuribara et al. 2006), it has been presumed to function within the cilia. However, its role in ciliogenesis and ciliary motility remains unclear. Therefore, in this study, we examined a phenotype of *Tetrahymena* cells lacking tArp.

## 2 Materials and Methods

### 2.1 *Tetrahymena* Strains and Culture Conditions

The *T. thermophila* strains used in this study are listed in Table S1. Most of these cell strains were provided by the Tetrahymena Stock Center. Cells were grown without shaking at 30°C in SPP medium (2% proteose peptone, 0.1% yeast extract, 0.2% glucose, and 0.003% Fe (III)‐EDTA). Live‐cell fluorescence imaging was also performed in SPP medium at 30°C. Starvation‐induced conjugation was conducted in 10 mM Tris–HCl (pH 7.5).

### 2.2 Construction of the EGFP–tArp Strains

An overview of the construction of the plasmid used for gene introduction is shown in Figure S1. The constructed plasmid was digested with SalI and KpnI, purified using a FastGene Gel/PCR Extraction Kit (NIPPON Genetics), and coated onto gold particles (0.6 μm in diameter, Bio‐Rad). DNA‐coated gold particles were introduced into WT (B2086 strain) cells that had been starved overnight in 10 mM Tris–HCl, using a biolistic gun PSD‐1000/He (Bio‐Rad) equipped with a 900‐psi rupture disk. Transformed cells were cultured in SPP medium supplemented with 1 μg/mL CdCl_2_ and 100 μg/mL paromomycin to select cells carrying the introduced DNA. To increase the percentage of recombinant DNA in the macronuclear genome, the concentration of CdCl_2_ was gradually reduced and the concentration of paromomycin was increased. Under conditions of 1 pg/mL CdCl_2_ and 2 mg/mL paromomycin, the macronuclear *tARP* gene was completely replaced with EGFP–*tARP* (Figure S2A). This strain was used in localization analyses and shutdown experiments.

### 2.3 Gene KO

Using genomic DNA from the WT strain (B2086) as a template, an approximately 1.3‐kb fragment from the 5′ flanking region of the *tARP* gene and a 1.4‐kb fragment from within the *tARP* gene were amplified by PCR with the primer pairs 9, 10 and 11, 12, respectively (Table S2, Figure S3). Each amplified fragment was cloned into the pT7blue vector (Merck Millipore) and subsequently inserted into the corresponding restriction enzyme sites of pNeo4 (provided by Dr. Mochizuki; Mochizuki 2008) to obtain the gene KO construct (Figure S3). The plasmid was then digested with SacI and KpnI and introduced into *T. thermophila* cells using the germline gene KO method (Dave et al. 2009). After isolating the G1 transformant, in which the *tARP* gene on chromosomes in the micronucleus was heterozygously disrupted, the resulting G1 cells were crossed with the Star strain (A*). As a result, we obtained several G2 cells in which the *tARP* allele in the micronucleus was homozygously disrupted. To obtain cells lacking the *tARP* gene in micro‐ and macronucleus, the G2 cells of different mating types were mated, and knockout cells were selected with paromomycin. The single cells were isolated and separately grown in the presence of paromomycin, and each clone was analyzed by PCR to confirm the correct insertion of the Neo4 cassette at the target locus.

### 2.4 Analysis of Cell Motility

Log‐phase cells were used in the analysis of cell motility. Swimming behavior was recorded at 7.5 frames per second (fps) using a DFK 21 AU618 camera (The Imaging Source). Recorded images were processed with the Z Project function in ImageJ (Schneider et al. 2012), and eight consecutive frames were merged to trace the swimming trajectory for approximately 1 s. The length of each swimming trajectory was measured using the line tool in ImageJ, and swimming velocity was calculated. The ciliary movement of somatic cilia was recorded at 1000 or 2000 fps using a HAS‐U2 high‐speed camera (DITECT) with a 20× objective lens. Using the images acquired at 1000 fps, the duration of a single ciliary beat cycle was measured to assess ciliary beat frequency. The effective stroke angle was measured from 2000 fps recordings using the angle tool in ImageJ.

### 2.5 Immunofluorescence Staining

Cells were washed twice with 20 mM phosphate buffer (pH 7.5) (PB) and fixed for 30 min in PB containing 2% paraformaldehyde. Fixed cells were then washed three times with PBS (137 mM NaCl, 2.7 mM KCl, 10 mM Na_2_HPO_4_, and 1.8 mM KH_2_PO_4_, pH 7.4) to remove the fixing solution. Permeabilization was performed using PBS containing 1% Triton X‐100, followed by three additional washes with PBS. The cells were resuspended in PBS containing 1% skim milk (PBS + S) for blocking. They were then incubated at 4°C overnight in PBS + S with the primary antiserum TAT1 (Woods et al. 1989) for microtubule staining, diluted 1:200. After three washes with PBS + S, the cells were incubated in PBS + S with a goat anti‐mouse IgG (H + L) highly cross‐adsorbed secondary antibody conjugated with Alexa Fluor 568 (Invitrogen), diluted 1:1000 at 4°C for 2 h. Cells were washed three times with PBS and observed under a fluorescence microscope.

### 2.6 Phylogenetic Analysis

The amino acid sequences of actin and Arps from 97 eukaryotic species, primarily focusing on SAR, were collected from GenBank (https://blast.ncbi.nlm.nih.gov/Blast.cgi), EukProt v3 (Richter et al. 2022; https://evocellbio.com/eukprot/), and Ciliate.org (https://ciliates.org/) (Table S3). Sequence collection was performed using BLASTp with the amino acid sequences of actin, actin isoforms, and each Arp from *H. sapiens* and *T. thermophila* as query sequences. Exact duplicates were removed using the rmdup command of SeqKit (Shen et al. 2016), and further redundancy was reduced using CD‐HIT with a sequence identity cut‐off of 0.9 (Li and Godzik 2006; Li et al. 2001, 2002). Multiple sequence alignment was performed with MAFFT ver. 7 using the L‐INS‐i algorithm (Katoh and Standley 2013). Regions with excessive gaps were trimmed using TrimAl (−gt 0.8) (Capella‐Gutiérrez et al. 2009). A maximum‐likelihood phylogenetic analysis was conducted with IQ‐TREE (Nguyen et al. 2015) using the best‐fit model selected by ModelFinder (LG + F + R10), and the branch supports of the resulting phylogenetic tree were estimated using UFboot (Hoang et al. 2018; Minh et al. 2013). The phylogenetic tree was inferred as unrooted. However, for descriptive purposes, we refer to the distinct clusters as ‘clades’ in this article.

### 2.7 Statistical Analysis

Statistical analyses were performed using R (R Core Team 2025). Statistical comparisons were conducted using the Steel–Dwass test implemented in the NSM3 package (Schneider et al. 2024). The significance of differences was set at *p* < 0.01. Boxplots and bar plots with individual data points were generated using the tidyverse (Wickham et al. 2019) and ggbeeswarm (Clarke et al. 2023) packages.

## 3 Results

### 3.1 tArp Is Widely Conserved in SAR

**FIGURE 1:**
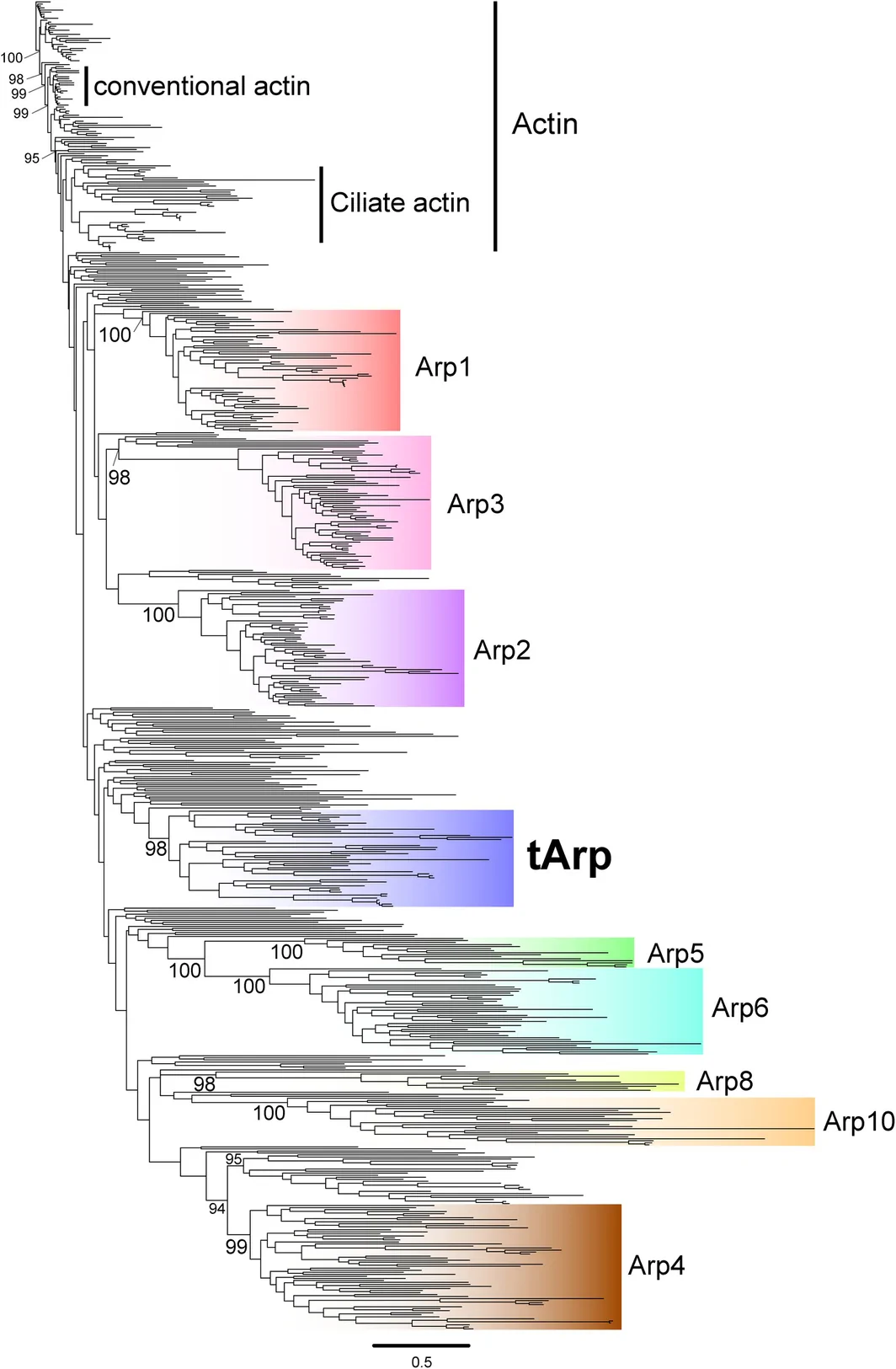
Maximum‐likelihood phylogenetic tree of Actin and Actin‐related proteins. Maximum‐likelihood phylogenetic tree of Actin and Arps from diverse eukaryotic lineages including SAR. The query sequences of Actin and each Arp formed distinct clades with well‐supported values. The original tree file is provided as Data S1.

**FIGURE 2:**
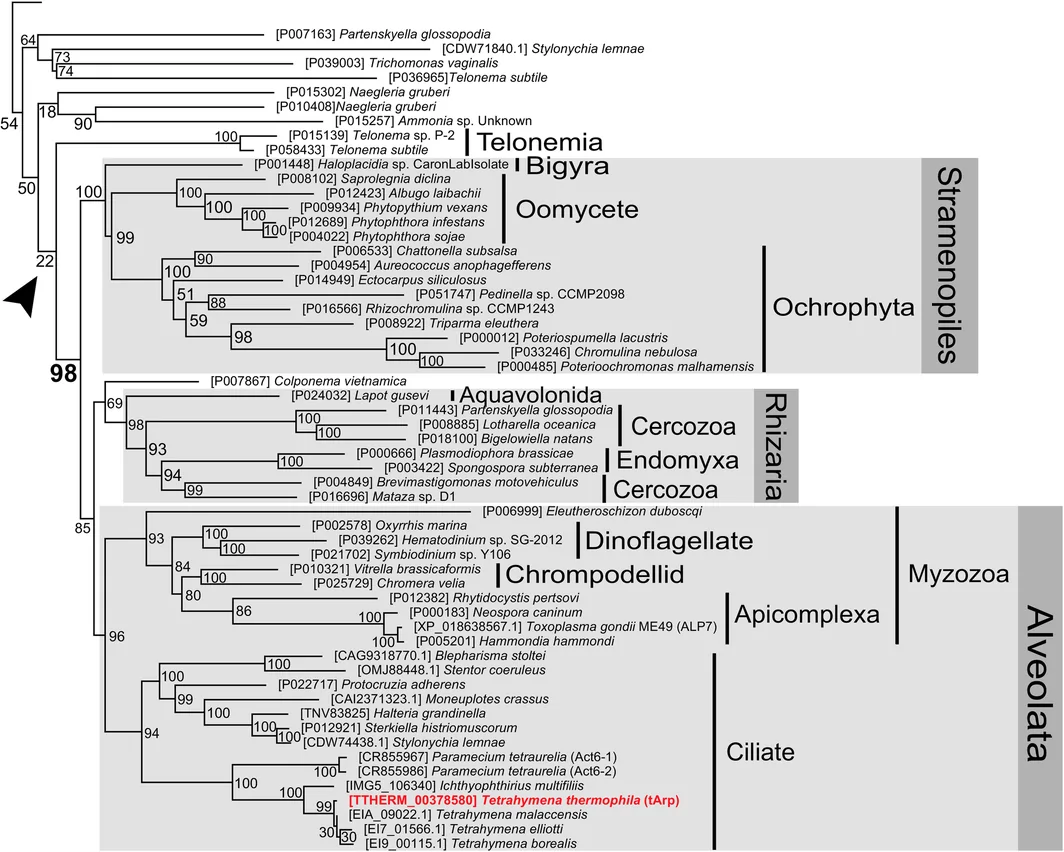
The clade containing *Tetrahymena* tArp and its neighboring branches. An enlarged view of the clade containing *Tetrahymena* tArp (indicated by bold red) and its neighboring branches in Figure 1. The tArp‐containing clade was strongly supported (UFBP = 98%) and consisted exclusively of sequences from species belonging to the SAR group. The clade of the sequences from Telonemia branched at the base of the tArp‐containing clade; however, the sister relationship between the two clades was weakly supported (UFBP = 22%; highlighted by a black arrowhead).

The ciliate *Tetrahymena*, from which tArp was identified initially, belongs to Alveolata. Recent advances in the tree of eukaryotes revealed that Alveolata shares the common ancestry exclusively with Stramenopiles and Rhizaria, and they are collectively termed SAR (Keeling and Burki 2019; Grattepanche et al. 2018; Adl et al. 2012). We investigated whether tArp, previously known only in *T. thermophila* , is conserved in closely related organisms. We, in particular, focused on species included in SAR and selected 97 species representing diverse eukaryotic lineages. Of these, 72 are of SAR lineage. We collected sequences predicted to be actin or Arp from GenBank (https://blast.ncbi.nlm.nih.gov/Blast.cgi), EukProt v3 (https://evocellbio.com/eukprot/), and Ciliate.org (https://ciliates.org/) by performing BLASTp searches using the amino acid sequences of *H. sapiens* and *T. thermophila* actin and all Arp proteins as query sequences (Table S3). A maximum‐likelihood (ML) phylogenetic analysis of the sequences inferred a tree in which the query actin and each Arp subfamily, including Arp1 ~6, 8, 10, were distributed into distinct clades, each supported by ultrafast bootstrap (UFBP) values of 98%–100% (Figure 1; Data S1). In this phylogenetic tree, actin and the known Arp families both formed generally monophyletic clades comprising sequences from diverse eukaryotic lineages. This result is consistent with previous molecular phylogenetic analyses of Arp (Morozov et al. 2018; Meagher et al. 2009; Sehring, Mansfeld, et al. 2007; Gordon and Sibley 2005; Kandasamy et al. 2004), indicating that our phylogenetic analysis was well balanced. The clade containing *Tetrahymena* tArp was composed of sequences exclusively from species belonging to SAR (Figure 2). This phylogenetic relationship was supported by a UFBP of 98%. This result suggests that tArp orthologs are conserved in many species belonging to SAR; tArp orthologues were detected in 47 of the 72 species belonging to SAR investigated in our phylogenetic analysis (Table S3). However, no orthologs of tArp were found in the remaining species. Although the absence of tArp orthologues in some SAR species could reflect incomplete genome data, it was not detected in well‐characterized lineages such as some apicomplexan species and diatoms, suggesting a genuine loss. These species are known to have lost their cilia and flagella or have markedly different ciliary and flagellar structures (Safonov 2024; Morrissette et al. 2023; Tandel et al. 2019; Nanjappa et al. 2017; Talman et al. 2014; Idei et al. 2013; Sinden et al. 2010; Wickstead and Gull 2007) (see Discussion in Data S2). This correlation found in SAR between the presence of tArp orthologs and the existence and structure of cilia may reflect the importance of tArp function in cilia. It is possible that the ancestral tArp existed in the common ancestor of SAR, and that secondary loss subsequently occurred in some species in association with the disappearance of cilia or flagella, or with the evolution of species‐specific cilia functions or structures.

### 3.2 tArp Is Present in Cilia

**FIGURE 3:**
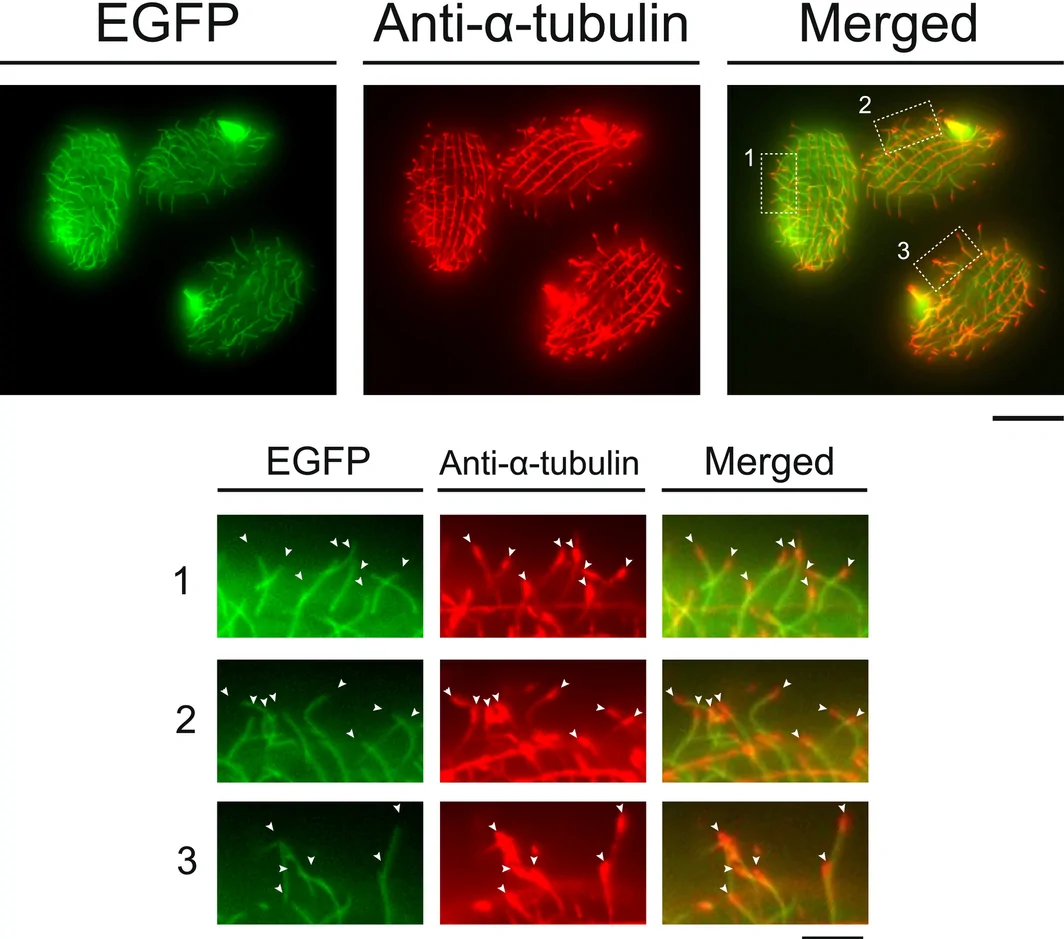
tArp specifically localizes to cilia. EGFP‐tArp expressing cells were cultured overnight in SPP medium supplemented with Cu^2+^ and then chemically fixed. Cells were immunostained with an anti–α‐tubulin antibody and observed by fluorescence microscopy. EGFP fluorescence was observed directly, without staining with anti‐GFP antibodies. Scale bar, 20 μm. The region outlined by the dashed box is shown at a higher magnification below. EGFP–tArp fluorescence was absent at the distal tip of the cilium (white arrowheads). Scale bar, 5 μm.

After expressing EGFP‐tArp in *T. thermophila* , the protein localized specifically to cilia (Figure 3 and Figure S2B,C). Based on the results of the shutdown experiments described below, the fusion with EGFP did not appear to significantly impair the intrinsic activity of tArp. Comparisons of the distribution of EGFP‐tArp and α‐tubulin within cilia revealed regions at the ciliary tip where EGFP‐tArp fluorescence was almost undetectable, with only α‐tubulin staining being present (Figure 3, magnified images). The length of this region was 1.09 ± 0.33 μm (mean ± SD, *n* = 150 cilia from 25 cells). Differences in the fluorescence distribution between axonemal proteins and tubulin have been reported in several studies (Bazan et al. 2021; Urbanska et al. 2018). Eukaryotic cilia are generally composed of a 9 + 2 axonemal structure; however, this organization is different at the distal tip of cilia (Legal et al. 2023; Soares et al. 2019; Louka et al. 2018; Reynolds et al. 2018; Fisch and Dupuis‐Williams 2011). Previous studies on *T. thermophila* estimated that the region containing only the central pair of microtubules was approximately 400–600 nm in length (Louka et al. 2018), and also that the length of the region in which B‐tubules were lost and only A‐tubules remained ranged from 0 to 1428 nm (Legal et al. 2023). The length of the region at the tip of the cilia where EGFP‐tArp fluorescence was undetectable appeared to be longer than the region where only central microtubules were observed. It is possible that tArp is present only in the region of the cilia where both A‐ and B‐tubules are present.

To further investigate the relationship between tArp and axonemal structures, we generated cells expressing tArp with EGFP fused to its C terminus under the control of the endogenous promoter (Figures S4 and S5). In this strain, tArp‐EGFP was observed in cilia (Figure S5B,C). We then performed a cilia fractionation assay and found that tArp‐EGFP was primarily detected in both the crude‐dynein fraction and outer‐doublet microtubule fraction (Figure S5D). The outer doublet microtubule fraction was further extracted using buffers with stepwise increases in the concentration of KCl, followed by extraction with 600 mM KI. The extraction of tArp was the most effective with 600 mM KCl. It is possible that tArp tightly bound to outer‐doublet microtubules.

### 3.3 tArp Is Essential for Proper Ciliary Movement

**FIGURE 4:**
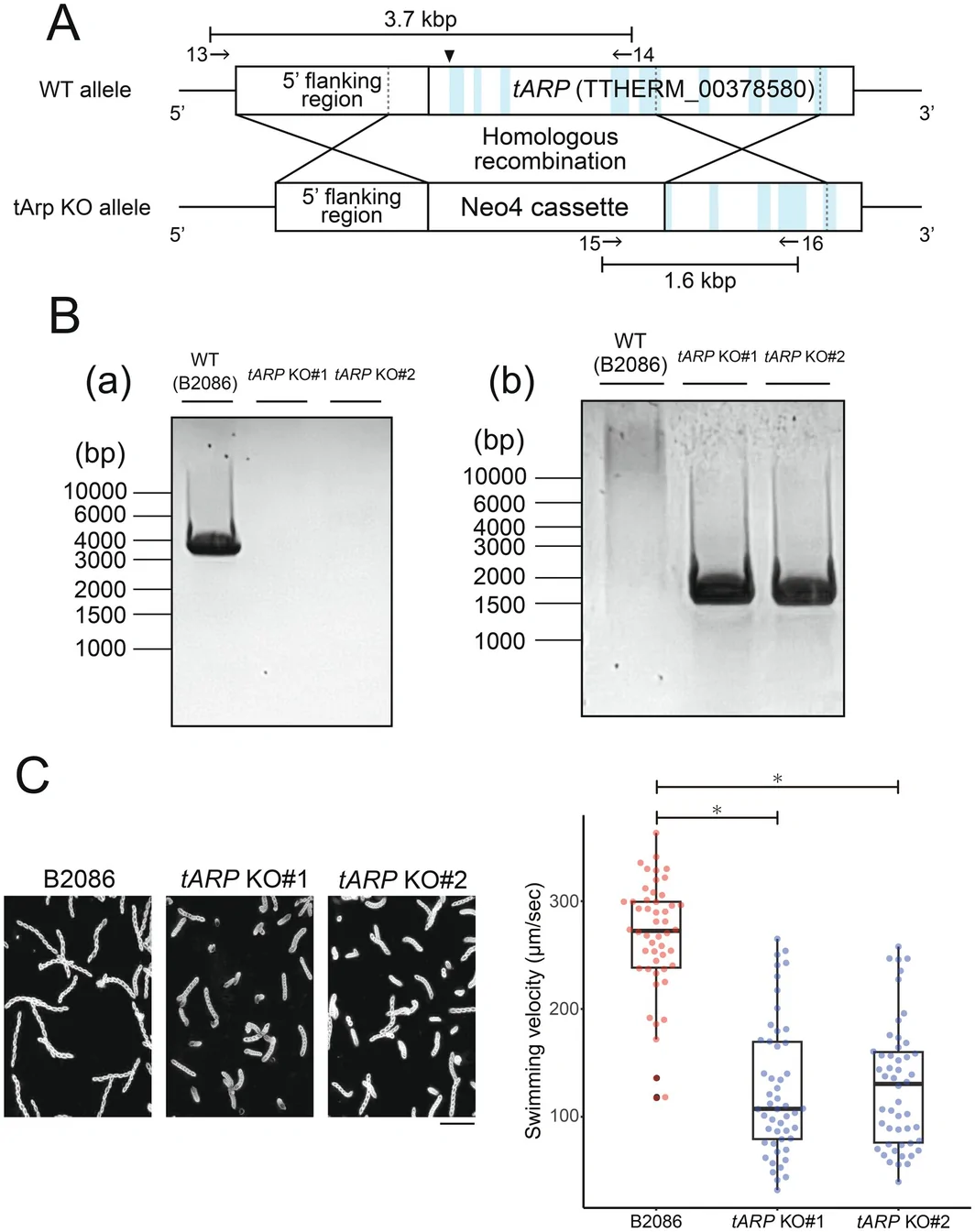
Swimming velocity is reduced in *tARP* KO cells. (A) Schematic of the *tARP*‐gene knockout construct. Non‐coding regions and exons of the *tARP* gene are shown in white and blue, respectively. The black triangle indicates the position of the translation initiation codon. A region of approximately 1.8‐kb downstream of the translation initiation codon was replaced with the paromomycin resistance marker, Neo4 cassette, to disrupt the gene. (B) Confirmation of gene disruption by PCR. (a) By using primers 13 and 14 (Table S2), an approximately 3.7‐kb fragment was amplified from the WT (B2086) genome, whereas no amplification was observed in the *tARP* KO allele. (b) By using primers 15 and 16 (Table S2), an approximately 1.6‐kb fragment containing part of the Neo4 gene and the 3′ end of the *tARP* ORF was amplified in only the *tARP* KO strains. (C) Representative swimming trajectories (1 s) of each strain. B2086 was used as the wild‐type strain. Trajectories were visualized by overlaying sequential images of swimming cells incubated at 30°C (left). Scale bar, 0.2 mm. Quantification of the swimming velocity of cells (right). *p* < 0.001, the Steel–Dwass test, *N* = 50 cells.

To investigate the cellular function of tArp, we prepared *tARP* KO strains in *T. thermophila* by germline gene knockout. Two individual cells were arbitrarily selected from the progeny and isolated, and the resulting strains, *tARP* KO#1 and *tARP* KO#2, were used in experiments (Figure 4). We initially examined the motility of *tARP* KO cells. The results obtained showed that swimming velocity was significantly lower in *tARP* KO cells than in wild‐type (WT) cells (WT: 293.5 ± 60.5 μm/s, *tARP* KO#1: 138.9 ± 69.0 μm/s, 143.1 ± 64.3 μm/s, mean ± SD, *n* = 50 cells) (Figure 4C). Furthermore, upon suppressing EGFP‐tArp expression from the *MTT2* promoter (Boldrin et al. 2008), which is specifically induced in the presence of copper ions, in the EGFP‐tArp strain, a decrease in swimming velocity was observed (Figure S6). We then analyzed ciliary beat frequency and the effective stroke angle in *tARP* KO cells using high‐speed camera imaging. The average beat frequency was approximately 40% lower in *tARP* KO cells than in WT cells, and abnormalities were also observed in the effective stroke angle (Figure S7, Videos [Link], [Link], [Link], [Link], [Link]). No significant differences were observed in the arrangement, length, or ability to form cilia between the *tARP* KO strains and WT (Figure S8, see Data S2). Therefore, the slower swimming velocity of *tARP* KO cells was primarily attributed to a decreased ciliary beat frequency and abnormal effective stroke angle. Similar phenotypes have been observed in mutants with impaired axonemal dynein function (Urbanska et al. 2018; Subramanian et al. 2016; Wood et al. 2007; Ludmann et al. 1993; Attwell et al. 1992) (see Figure S7).

## 4 Discussion

In the present study, we demonstrated that tArp is not a species‐specific protein, but a member of a novel Arp family spanning the eukaryotic supergroup SAR. Furthermore, gene disruption experiments revealed the importance of tArp for maintaining a normal ciliary beat frequency and effective stroke angle in *T. thermophila* . In our phylogenetic analysis, Act6‐1 and Act6‐2 in the other ciliate *Paramecium tetraurelia*, belonging to the Oligohymenophorea subphylum as *Tetrahymena*, were shown to be monophyletic with tArp (Figure 2). A proteome analysis revealed that Act6‐1 and Act6‐2 were both present in cilia (Arnaiz et al. 2009). Large‐scale functional protein analyses of actin and Arp have been conducted on *P. tetraurelia* (Sehring, Reiner, et al. 2007). However, GFP‐Act6‐1 fluorescence was only detected in the cytoplasm, and no noticeable phenotype was observed following the double knockdown of their genes. Actin and actin‐related proteins appear to be more diverse in *Paramecium* than in *Tetrahymena* (Arnaiz et al. 2009). Further analyses are needed to determine whether the functions of tArp‐family proteins in ciliary motility differ between *Tetrahymena* and *Paramecium*. In addition, gene manipulation methods have recently been explored for dinoflagellates (Gornik et al. 2022; Sprecher et al. 2020; Ortiz‐Matamoros et al. 2015), its closely related species *Perkinsus marinus* (Sakamoto et al. 2022), and Stramenopiles (Poveda‐Huertes et al. 2023), which may provide a more detailed understanding of the conservation of the cellular function of tArp in SAR in the future.

It remains unclear whether the phylogenetic distribution of tArp is limited to species belonging to SAR. According to recent phylogenetic analyses of multiple proteins, Telonemia and Haptista are the candidate lineages closely related to SAR. Recent phylogenomic analyses recovered the sister relationship between Telonemia and SAR, invoking a further larger assemblage, so‐called “TSAR” (Valt et al. 2025; Yazaki et al. 2025; Strassert et al. 2019). Nevertheless, other phylogenomic analyses united Telonemia, Haptophyta, and Centrohelida into a clade, which was further connected directly to SAR (Torruella et al. 2025; Zlatogursky et al. 2025). In the phylogenetic tree shown in Figure 2, the Telonemia sequences were placed at the base of the tArp clade, albeit this particular relationship received a negligible UFBP as low as 22%. The sequences identified in haptophytes and centrohelids were distantly related to the tArp clade (Figure 2, Data S1). Based on the results described above, we tentatively conclude that the tArp ortholog is restricted only to the species belonging to SAR. To examine this conclusion, we first need to pinpoint the close relatives of SAR in the tree of eukaryotes, in particular the position of Telonemia relative to SAR.

Recent cryo‐electron microscopy analyses showed that actin binds to the tail region of single‐head inner arm dynein, associated with p28 or centrin, in humans, *Chlamydomonas*, and *Trypanosoma* (Xia et al. 2025; Walton et al. 2023). In *T. thermophila* , biochemical experiments revealed that actin co‐sedimented with the 14S dynein fraction (Muto et al. 1994), which appeared to be single‐head inner arm dynein (Marchese‐Ragona et al. 1988; Toyoshima 1987). In addition to conventional actin, tArp was detected not only in the outer‐doublet microtubule fraction, but also in the crude‐dynein fraction (see Figure S5D). *T. thermophila* possesses as many as 18 highly diversified single‐head inner arm dynein heavy chains (Wilkes et al. 2008). Inner arm dynein generally controls the waveform of ciliary movement (Yamamoto et al. 2021). However, a reduced ciliary beat frequency has been reported in cells lacking *DYH8* or *DYH11*, which encode the single‐headed inner arm dynein, in *T. thermophila* (Subramanian et al. 2016; Liu et al. 2004). In addition, three distinct p28 proteins have been identified in *T. thermophila* , and each likely plays an important role in ciliary motility by acting on different inner arm dyneins (Subramanian et al. 2016; Wilkes et al. 2007). A proteome‐wide cross‐linking analysis of cilia in this organism recently revealed that tArp may be located in close proximity to the single‐headed inner arm dynein DYH15 (McCafferty et al. 2025). Therefore, further biochemical analyses will be important for elucidating the relationships among tArp, highly diversified single‐headed inner arm dyneins, including DYH15, and their associated proteins, such as diversified p28 proteins, in *T. thermophila* .

Comparisons of the functions of actin and tArp in cilia may provide insights into the significance of the acquisition of tArp, in addition to actin, by the ancestral lineage of SAR. As far as we know, no ciliary proteins have been reported that are conserved uniquely across all major SAR lineages. Therefore, the identification of the tArp family may provide new insights into the evolution and diversification of ciliary structures in SAR.

## Funding

This work was supported by the Japan Society for the Promotion of Science (JSPS) KAKENHI grants (23K05746).

## Supporting information


**Table S1:** jeu70123‐sup‐0001‐TableS1.xlsx. *Tetrahymena thermophila* strains used in this study.

**Table S2:** PCR primers used in this study.

**Table S3:** List of species used in the phylogenetic analysis in this study. List of the 97 eukaryotic species used for phylogenetic analysis. Species in which tArp was identified are indicated with “+” in the “tArp presence” column, whereas species in which tArp was not found are indicated with “nf (not found).”

**Figure S1:** Construction of the EGFP‐tArp construct. Using genomic DNA from the B2086 strain as a template, a region of approximately 2.4‐kb encompassing approximately 1.4‐kb upstream and 1.0‐kb downstream of the start codon (black triangle) of the *tARP* gene was amplified by PCR using primers 1 and 2 (Table S2). This fragment was cloned into the pT7Blue vector (Merck Millipore) to generate the pT7Blue–tARP vector. Using the pT7Blue–tARP vector as a template, an inverse PCR product was amplified with primers 5 and 6 (Table S2). Moreover, the Neo4–MTT2–EGFP was amplified using primers 3 and 4 (Table S2). These two DNA fragments were fused by an In‐Fusion reaction to generate the final construct used in the experiment shown in Figure S2. The non‐coding regions and exons of *tARP* are shown in white and blue, respectively. The Neo4 cassette is derived from pNeo4 kindly provided by Dr. Mochizuki and is composed of the *MTT1* promoter + neo4 + 3′BTU2 terminator (Mochizuki 2008).

**Figure S2:** Generation of the EGFP–tArp strain and analyses of its expression and cellular localization. (A) Schematic of the EGFP–tArp expression construct (a). Non‐coding regions and exons of the *tARP* gene are shown in white and blue, respectively. Black triangles indicate the position of the start codon of *tARP*. Transformed cells were selected, and the complete replacement of all *tARP* genes on macronuclear chromosomes with the introduced construct was confirmed by PCR. By using primers 7 and 8 (Table S2), an approximately 2.7‐kb fragment was amplified from the wild‐type (WT) genome, whereas an approximately 6.7‐kb fragment was amplified from the isolated strain (b). (B) Fluorescence images of living EGFP–tArp strain cells. Cells cultured in SPP medium without CuSO_4_ showed a weak or no detectable EGFP signal, except for weak autofluorescence from food vacuoles. In some cells, faint fluorescence was observed at the oral apparatus (white arrow), at which cilia were densely packed. On the other hand, cells cultured with CuSO_4_ at a final concentration of 1.25 mM showed fluorescence throughout the cytoplasm and cilia. The dark region in the center of the cell corresponds to the location of the macronucleus. Scale bar, 20 μm. (C) Western blot analysis of the EGFP–tArp strain after dibucaine treatment. Cells before (lanes 1, 2) and after Cu^2+^‐induced expression (lanes 3, 4) were treated with dibucaine, separated into cell body (lanes 1, 3) and ciliary fractions (lanes 2, 4), and then subjected to SDS‐PAGE (CBB‐stained gel, left). A gel prepared under the same conditions was transferred to a membrane and subjected to Western blotting using an anti‐GFP antibody (right). Although a faint signal was detected in uninduced cells (lane 2), a clear band corresponding to the expected molecular weight of EGFP–tArp was detected in the ciliary fraction of induced cells (lane 4). The smaller bands visible in lane 3 most likely correspond to the products of EGFP‐tArp degradation.

**Figure S3:** Construction of the tArp knockout construct. Using genomic DNA from the B2086 strain as a template, an approximately 1.3‐kb fragment from the 5′ flanking region of the *tARP* gene and a 1.4‐kb fragment from within the *tARP* gene were amplified by PCR with primer sets 9 and 10 or 11 and 12 (Table S2), respectively. These primers contain SacI, PstI, ClaI, or XhoI recognition sites at their 5′‐ends, respectively. Each PCR product was cloned into a cloning vector and subsequently excised with the corresponding restriction enzymes. The fragments were then sequentially inserted into the pNeo4 plasmid to generate the final product. The non‐coding regions and exons of *tARP* are shown in white and blue, respectively. Black triangles indicate the position of the start codon of *tARP*.

**Figure S4:** Construction of tArp‐EGFP. Using genomic DNA from the B2086 strain as a template, a region of approximately 2.0 kb encompassing the stop codon (white arrowhead) of the *tARP* gene was amplified by PCR using primers 17 and 18 (Table S2). This PCR product was cloned into the pT7Blue vector (Merck Millipore) to generate the pT7Blue–*tARP_*Cter vector. Using it as a template, an inverse PCR product lacking the stop codon of *tARP* was amplified with primers 21 and 22 (Table S2). The EGFP‐Neo4 cassette (Kataoka et al., 2010) was amplified using primers 19 and 20 (Table S2). These two PCR products were fused by an In‐Fusion reaction to generate the final construct. The non‐coding regions and exons of *tARP* are shown in white and blue, respectively.

**Figure S5:** Generation of strains expressing tArp‐EGFP under the control of the endogenous promoter. (A) Schematic diagram of the tArp‐EGFP‐expression construct (a). The *tARP* gene locus on the macronuclear chromosomes of wild‐type cells (B2086) was replaced via homologous recombination with a gene fragment derived from the construct shown in Figure S4. Transformed cells were selected, and strains in which all *tARP* loci had been replaced by the construct were isolated. Using primers 23 and 24, a fragment of approximately 2.3 kb was amplified by PCR from the parent genome (left), while a fragment of approximately 5.4 kb was amplified from the isolated strain (right). (B) A fluorescence image of a living tArp‐EGFP cell. EGFP signal was observed in both somatic and oral cilia as indicated by white and magenta arrowheads, respectively. Scale bar, 20 μm. (C) Western blot of tArp‐EGFP. Cells were deciliated with dibucaine, and cell body (lane 1) and cilia (lane 2) proteins were analyzed by SDS‐PAGE. Both fractions were applied with the same ratio relative to the original cell. To the right, a corresponding Western blot with anti‐GFP antibodies. A strong band corresponding to the expected molecular weight of tArp‐EGFP was detected in the ciliary fraction (lane 2). (D) tArp‐EGFP was detected in both the crude‐dynein fraction and outer‐doublet microtubule fraction. Left: A CBB‐stained gel showing proteins separated by SDS‐PAGE from samples subjected to the fractionation experiment (see below); right: a Western blot of samples treated in the same manner using a GFP antibody. Lane 1, total proteins from isolated cilia; lane 2, the membrane‐matrix fraction; lane 3, the crude dynein fraction; lanes 4–8, fractions successively extracted from outer‐doublet microtubules using solutions containing 200, 400, 500, and 600 mM KCl, and 600 mM KI, respectively; lane 9, the insoluble pellet remaining after extraction with 600 mM KI.

**Figure S6:** Suppressing tArp expression reduced the swimming velocity of cells. (A) Representative swimming trajectories (1 s) of WT (B2086) and EGFP–tArp cells in the absence or presence of Cu^2+^ (final concentration, 1.25 mM) at 30°C. Trajectories were visualized by overlaying sequential images of swimming cells (1 s). Scale bar, 0.2 mm. (B) Quantification of the swimming velocity of cells. In the absence of Cu^2+^, swimming velocity was approximately 30% slower in EGFP–tArp cells than in WT cells. *p* < 0.001, the Steel–Dwass test, *N* = 150 cells. ns, not significant.

**Figure S7:** Ciliary beat frequency and effective stroke angle in *tARP* KO cells. (A) The ciliary beat frequencies of WT, *tARP* KO, *DYH6* KO, and *OAD* mutant cells were measured using a high‐speed camera at 1000 frames per second (fps). *p* < 0.001, the Steel–Dwass test, *N* = 120 cilia (20 cells). ns, not significant. (B) Effective stroke angles were identified based on ciliary motion recorded with a high‐speed camera at 2000 fps. *p* < 0.001, the Steel–Dwass test, *N* = 120 cilia (20 cells). ns, not significant. Axonemal dyneins play a central role in ciliary motility and are generally classified into outer and inner arm dyneins. Outer arm dyneins generally contribute to ciliary beat frequency, whereas inner arm dyneins are responsible for forming the ciliary wave pattern. *DYH6* KO6 cells lack the α heavy chain of the two‐headed inner arm (f/I1) dynein (Angus et al., 2001). The *OAD1 C11* mutant lacks outer arm dyneins and becomes immotile at a restrictive temperature of 38°C, whereas even at a permissive temperature, the partial loss of outer arm dyneins results in a slower swimming velocity than that by WT cells (Ludmann et al. 1993; Attwell et al. 1992). In *tARP* KO cells, ciliary beat frequency and the effective stroke angle were both reduced. All cells were cultured at 30°C.

**Figure S8:** The loss of tArp does not significantly affect ciliary distribution, length, or regeneration. (A) Immunofluorescence staining of α‐tubulin. Representative images of cells are shown. TAT1 and goat anti‐mouse IgG (H + L) conjugated with Alexa Fluor Plus 488 were used as the primary and secondary antibodies, respectively. Scale bar, 10 μm. (B) Ciliary length. Cilia removed from the cell body by the dibucaine treatment were collected by centrifugation. Ciliary length was measured under a microscope. The Steel–Dwass test, *N* = 150 cilia. ns, not significant. (C) Ciliary regeneration assay. *Tetrahymena thermophila* cells regenerate cilia after deciliation. The swimming trajectories of deciliated cells were traced over time following dibucaine‐induced deciliation. Immediately after deciliation, cells lost motility due to the absence of cilia. Motility gradually recovered over time. WT and *tARP* KO cells both recovered motility within approximately 3 h. Scale bar, 200 μm. All cells used in experiments were cultured at 30°C.

**Data S1:** Maximum‐likelihood phylogenetic tree of actin and Arps used in this study, as shown in Figure 1. The tree is provided in the Newick format, and OTU labels are shown in the format “organism name_accession number”.

**Data S2:** Materials and methods.

**Video S1:** High‐speed video recording of ciliary motion. B2086 cells were cultured at 30°C in SPP, and the ciliary movement was recorded at 2,000 fps using a high‐speed camera.

**Video S2:** High‐speed imaging of ciliary motion in *tARP* KO#1 cells. Experience was conducted in the same manner as in Video S1.

**Video S3:** High‐speed imaging of ciliary motion in *tARP* KO#2 cells. Experience was conducted in the same manner as in Video S1.

**Video S4:** High‐speed imaging of ciliary motion in *DYH6* KO6 cells. Experience was conducted in the same manner as in Video S1.

**Video S5:** High‐speed imaging of ciliary motion in *OAD1 C11* cells. Experience was conducted in the same manner as in Video S1.
