# Supplementary material for: tArp Is an Actin‐Related Protein Required for Ciliary Function That Is Conserved in Ciliates and Related Eukaryotic Groups
Source: J Eukaryot Microbiol. 2026 Sep 18;73(5):e70123. doi: 10.1111/jeu.70123 (PMC13589156; doi:10.1111/jeu.70123)
Supplement: Supplementary file 1 [file JEU-73-e70123-s002.pdf]

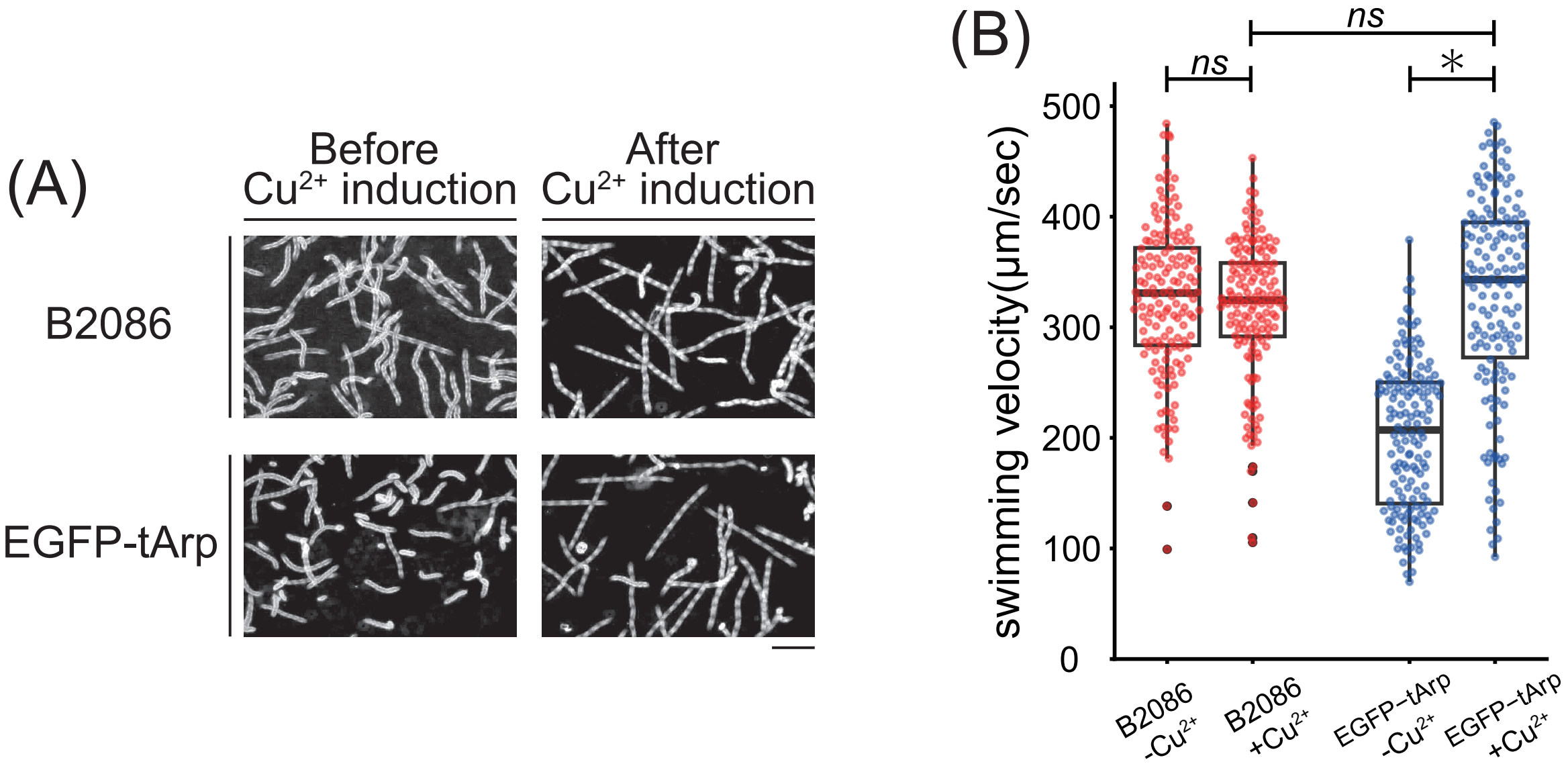

(A)
Before
Cu2+ induction
After
Cu2+ induction
B2086
EGFP-tArp
(B)
ns
ns
*
swimming velocity(μm/sec)
500
400
300
200
100
0
B2086
-Cu2+
B2086
+Cu2+
EGFP−tArp
-Cu2+
EGFP−tArp
+Cu2+
