# Supplementary material for: tArp Is an Actin‐Related Protein Required for Ciliary Function That Is Conserved in Ciliates and Related Eukaryotic Groups
Source: J Eukaryot Microbiol. 2026 Sep 18;73(5):e70123. doi: 10.1111/jeu.70123 (PMC13589156; doi:10.1111/jeu.70123)
Supplement: Supplementary file 2 [file JEU-73-e70123-s003.pdf]

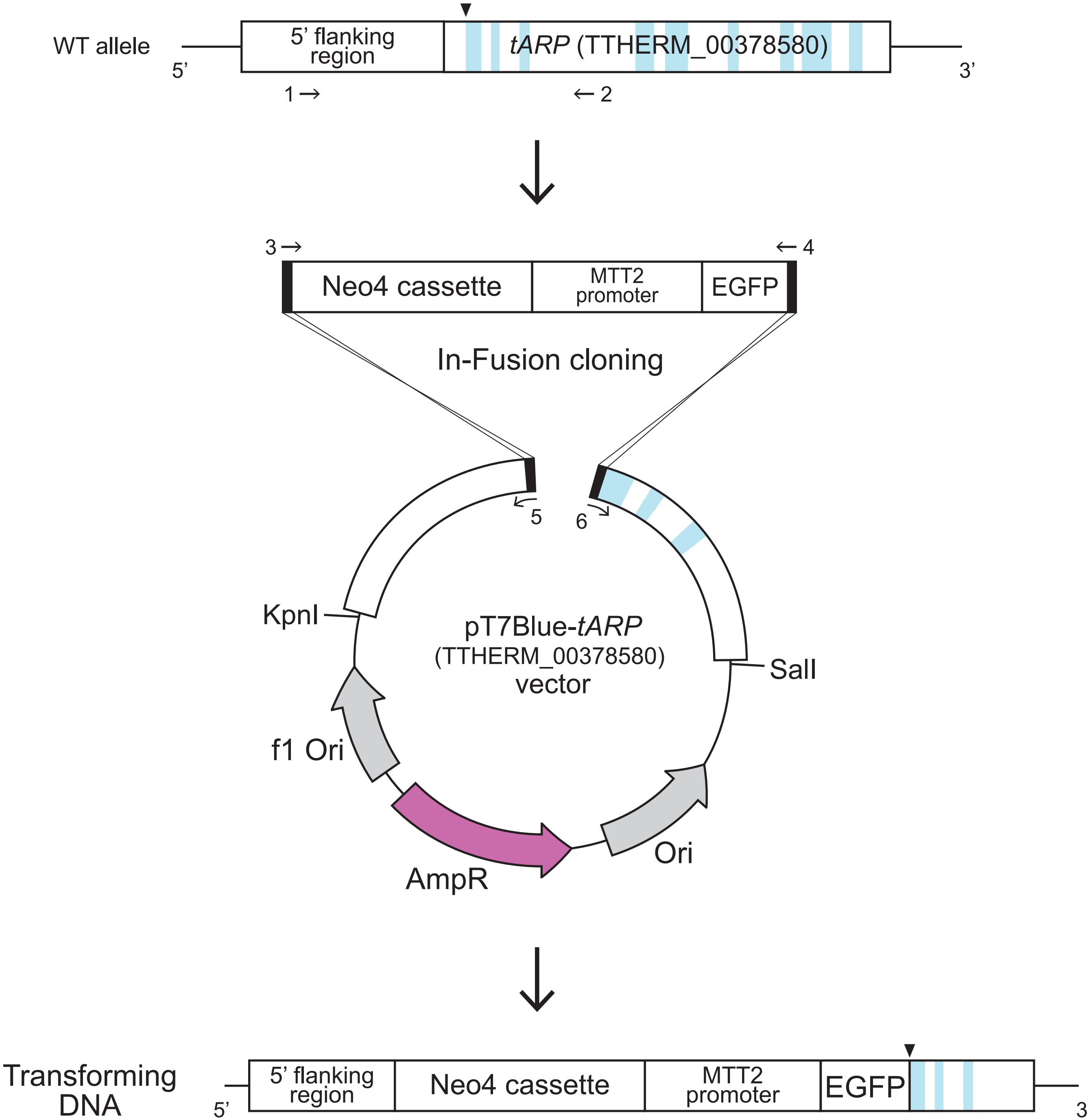
WT allele
5'
5' flanking region
tARP (TTHERM_00378580)
3'
1 →
← 2
3 →
← 4
Neo4 cassette
MTT2 promoter
EGFP
In-Fusion cloning
5
6
KpnI
pT7Blue-tARP (TTHERM_00378580) vector
SalI
f1 Ori
AmpR
Ori
Transforming DNA
5'
5' flanking region
Neo4 cassette
MTT2 promoter
EGFP
3'
