# Supplementary material for: tArp Is an Actin‐Related Protein Required for Ciliary Function That Is Conserved in Ciliates and Related Eukaryotic Groups
Source: J Eukaryot Microbiol. 2026 Sep 18;73(5):e70123. doi: 10.1111/jeu.70123 (PMC13589156; doi:10.1111/jeu.70123)
Supplement: Supplementary file 3 [file JEU-73-e70123-s005.pdf]

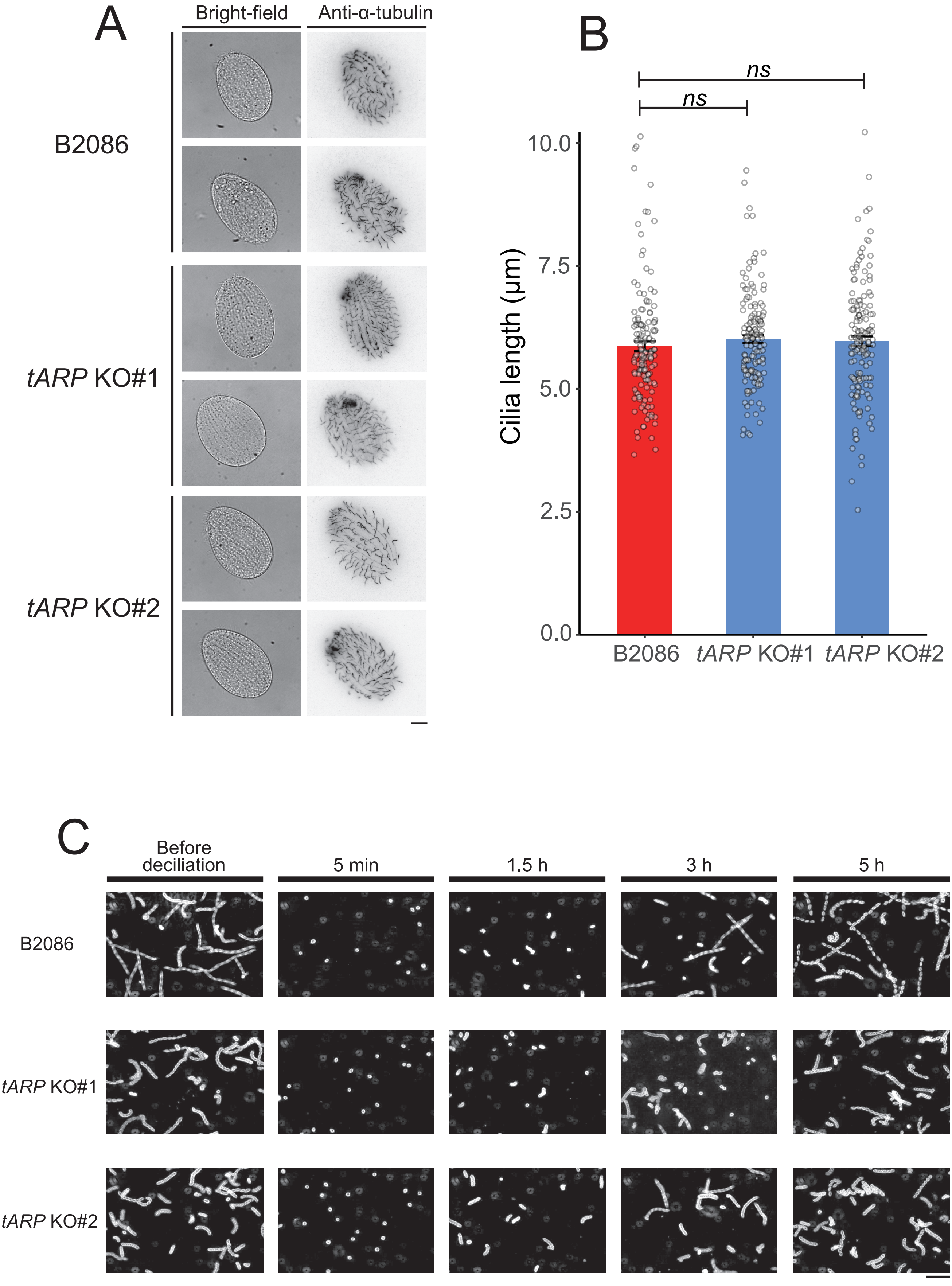
A
Bright-field
Anti-α-tubulin
B2086
tARP KO#1
tARP KO#2
B
ns
ns
Cilia length (µm)
10.0
7.5
5.0
2.5
0.0
B2086
tARP KO#1
tARP KO#2
C
Before deciliation
5 min
1.5 h
3 h
5 h
B2086
tARP KO#1
tARP KO#2
