# Supplementary material for: tArp Is an Actin‐Related Protein Required for Ciliary Function That Is Conserved in Ciliates and Related Eukaryotic Groups
Source: J Eukaryot Microbiol. 2026 Sep 18;73(5):e70123. doi: 10.1111/jeu.70123 (PMC13589156; doi:10.1111/jeu.70123)
Supplement: Supplementary file 4 [file JEU-73-e70123-s008.pdf]

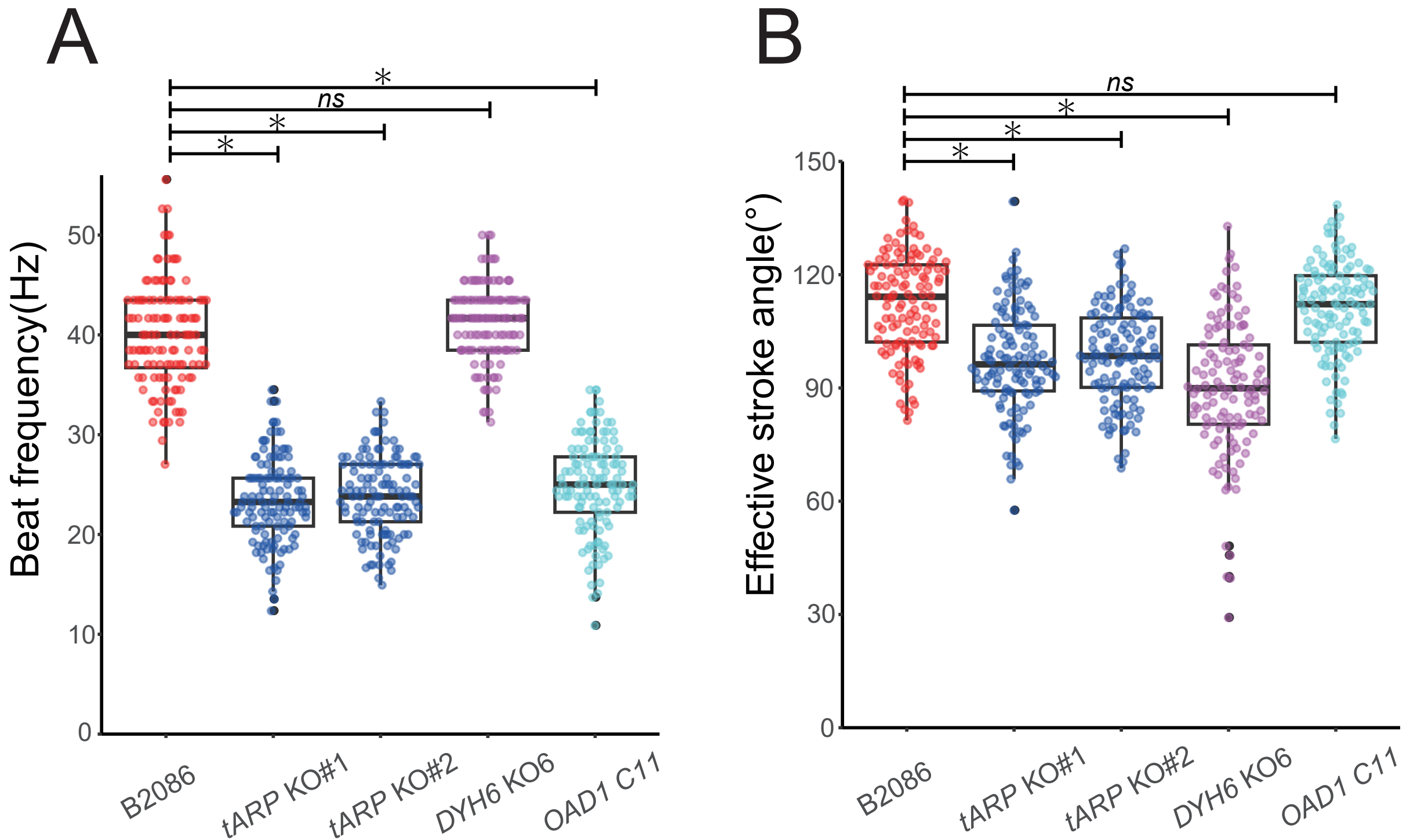

A
B
*
ns
*
*
Beat frequency(Hz)
0
10
20
30
40
50
B2086
tARP KO#1
tARP KO#2
DYH6 KO6
OAD1 C11
ns
*
*
*
Effective stroke angle(°)
0
30
60
90
120
150
B2086
tARP KO#1
tARP KO#2
DYH6 KO6
OAD1 C11
