# Supplementary material for: tArp Is an Actin‐Related Protein Required for Ciliary Function That Is Conserved in Ciliates and Related Eukaryotic Groups
Source: J Eukaryot Microbiol. 2026 Sep 18;73(5):e70123. doi: 10.1111/jeu.70123 (PMC13589156; doi:10.1111/jeu.70123)
Supplement: Supplementary file 5 [file JEU-73-e70123-s010.pdf]

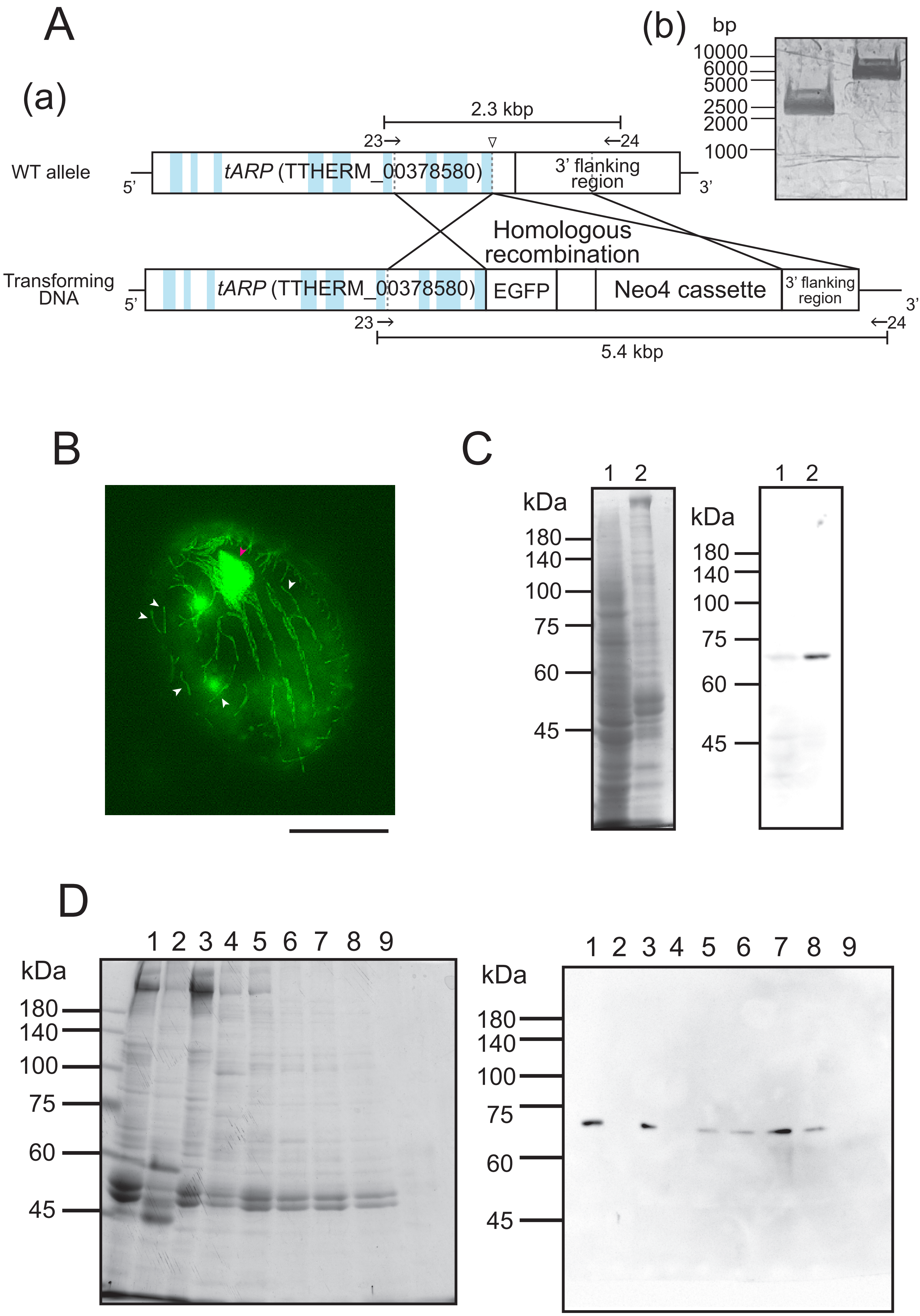

A
(a)
(b)
bp
10000
6000
5000
2500
2000
1000
2.3 kbp
23→
←24
WT allele
5'
3'
tARP (TTHERM_00378580)
3' flanking region
Homologous recombination
Transforming DNA
EGFP
Neo4 cassette
5.4 kbp
B
C
1
2
kDa
180
140
100
75
60
45
D
1 2 3 4 5 6 7 8 9
