# Supplementary material for: tArp Is an Actin‐Related Protein Required for Ciliary Function That Is Conserved in Ciliates and Related Eukaryotic Groups
Source: J Eukaryot Microbiol. 2026 Sep 18;73(5):e70123. doi: 10.1111/jeu.70123 (PMC13589156; doi:10.1111/jeu.70123)
Supplement: Supplementary file 6 [file JEU-73-e70123-s011.pdf]

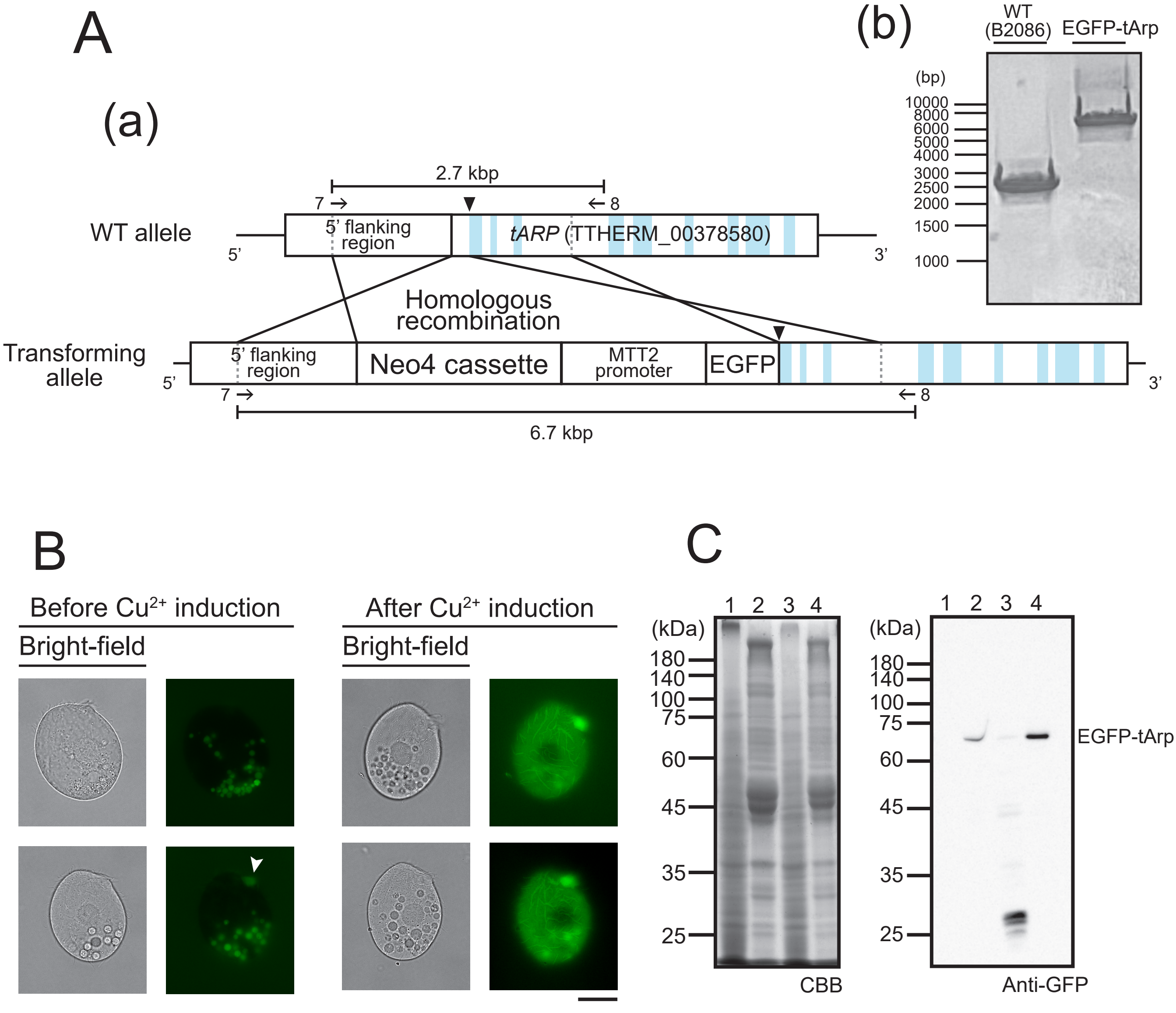
A
(a)
2.7 kbp
7 →
← 8
WT allele
5' flanking region
tARP (TTHERM_00378580)
5'
3'
Homologous recombination
Transforming allele
5' flanking region
Neo4 cassette
MTT2 promoter
EGFP
5'
3'
7 →
← 8
6.7 kbp
(b)
WT (B2086)
EGFP-tArp
(bp)
10000
8000
6000
5000
4000
3000
2500
2000
1500
1000
B
Before Cu2+ induction
Bright-field
After Cu2+ induction
Bright-field
C
1 2 3 4
(kDa)
180
140
100
75
60
45
35
25
CBB
1 2 3 4
(kDa)
180
140
100
75
60
45
35
25
EGFP-tArp
Anti-GFP
