# Supplementary material for: tArp Is an Actin‐Related Protein Required for Ciliary Function That Is Conserved in Ciliates and Related Eukaryotic Groups
Source: J Eukaryot Microbiol. 2026 Sep 18;73(5):e70123. doi: 10.1111/jeu.70123 (PMC13589156; doi:10.1111/jeu.70123)
Supplement: Supplementary file 7 [file JEU-73-e70123-s012.pdf]

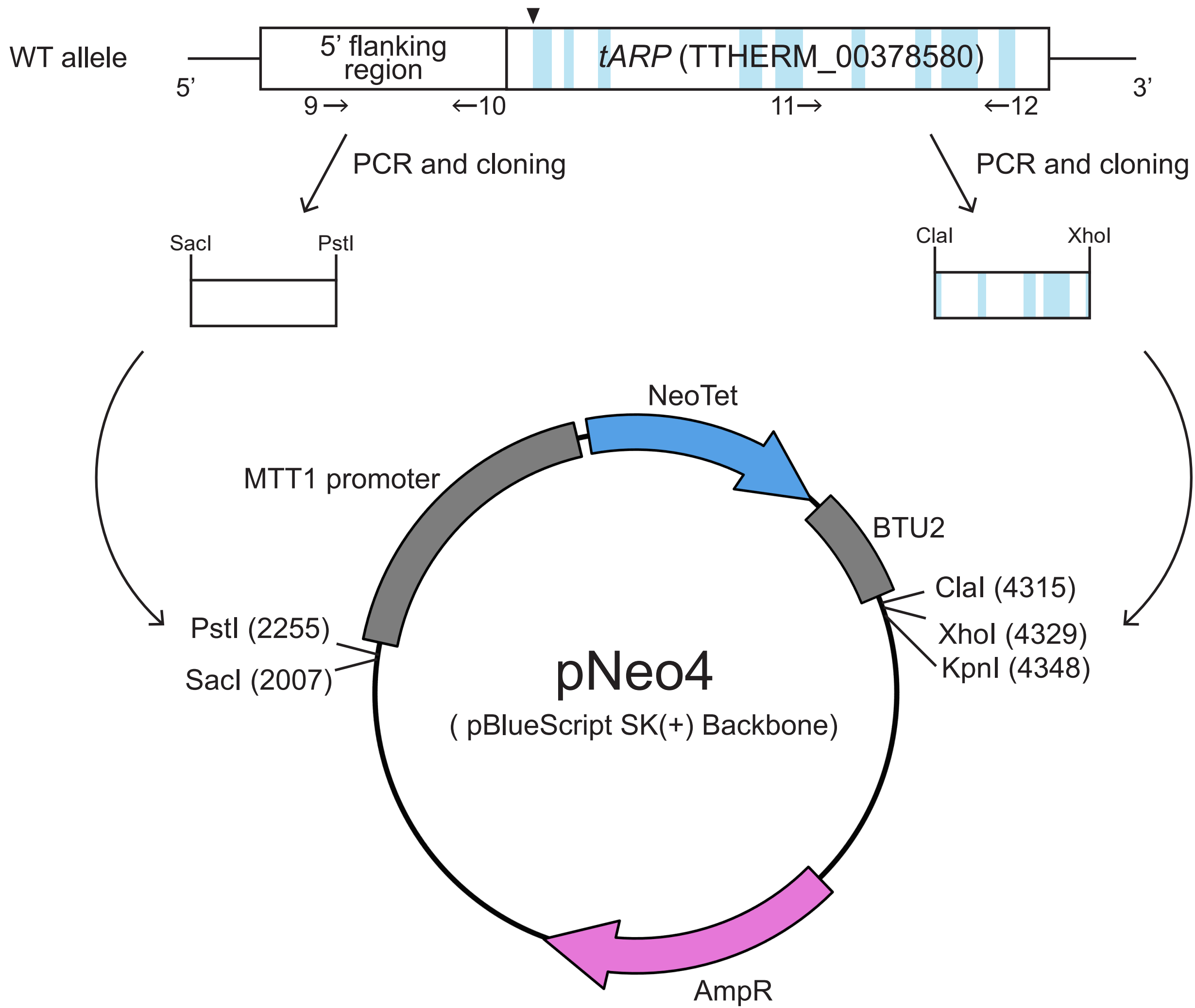
WT allele
5'
5' flanking region
tARP (TTHERM_00378580)
3'
9 →
←10
11→
←12
PCR and cloning
PCR and cloning
SacI
PstI
ClaI
XhoI
NeoTet
MTT1 promoter
BTU2
ClaI (4315)
XhoI (4329)
KpnI (4348)
PstI (2255)
SacI (2007)
pNeo4
(pBlueScript SK(+) Backbone)
AmpR
