# Supplementary material for: tArp Is an Actin‐Related Protein Required for Ciliary Function That Is Conserved in Ciliates and Related Eukaryotic Groups
Source: J Eukaryot Microbiol. 2026 Sep 18;73(5):e70123. doi: 10.1111/jeu.70123 (PMC13589156; doi:10.1111/jeu.70123)
Supplement: Supplementary file 8 [file JEU-73-e70123-s014.pdf]

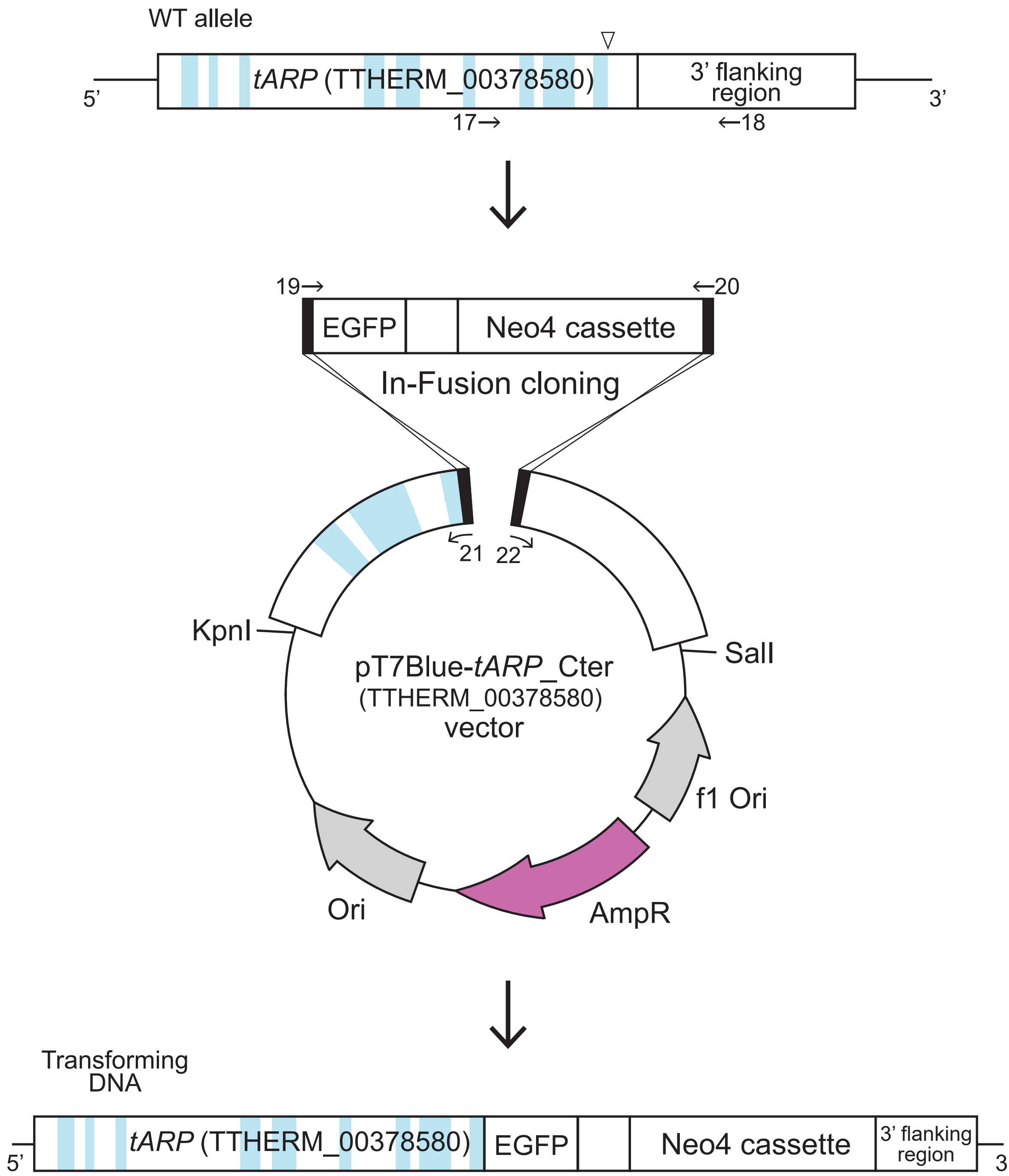
WT allele
5'
tARP (TTHERM_00378580)
3' flanking region
3'
17→
←18
19→
←20
EGFP
Neo4 cassette
In-Fusion cloning
21
22
KpnI
SalI
pT7Blue-tARP_Cter
(TTHERM_00378580)
vector
f1 Ori
AmpR
Ori
Transforming DNA
5'
tARP (TTHERM_00378580)
EGFP
Neo4 cassette
3' flanking region
3'
