# Supplementary material for: tArp Is an Actin‐Related Protein Required for Ciliary Function That Is Conserved in Ciliates and Related Eukaryotic Groups
Source: J Eukaryot Microbiol. 2026 Sep 18;73(5):e70123. doi: 10.1111/jeu.70123 (PMC13589156; doi:10.1111/jeu.70123)
Supplement: Supplementary file 9 [file JEU-73-e70123-s015.docx]

**Supplementary Materials and Methods**

**Construction of tArp-EGFP strains**

Figure S4 shows an overview of the construction of the plasmid used to generate tArp-EGFP strain. DNA fragments obtained by digesting the constructed plasmid with SalI and KpnI were purified using the FastGene Gel/PCR Extraction Kit (NIPPON Genetics) and then coated onto gold particles (0.6 µm in diameter, Bio-Rad). DNA-coated gold particles were introduced into WT (B2086 strain) cells that had been starved overnight in 10 mM Tris-HCl using a biolistic gun PSD-1000/He (Bio-Rad) equipped with a 900-psi rupture disk. Transformed cells were cultured in SPP medium supplemented with 1 µg/mL CdCl_2_ and 100 µg/mL paromomycin to select cells transformed with the introduced DNA. To increase the propotion of *tArp-EGFP* integrated into the macronuclear genome, the concentration of CdCl_2_ was gradually reduced, and that of paromomycin was increased to strengthen the selection pressure. Under conditions of 1 pg/mL CdCl_2_ and 1.2 mg/mL paromomycin, the macronuclear *tARP* gene was completely replaced with *tARP*–EGFP (Figure S5). This cell strain was cultured at 30°C in SPP medium and used to observe the localization of tArp.

**Deciliation of cell**

Dibucaine solution was added directly to log-phase cell cultures to a final concentration of 1.25 mM (Thompson et al., 1974). After confirming that nearly all cells in the field had ceased swimming, deciliated cells and released cilia were promptly separated using a hand centrifuge. Cilia present in the supernatant were collected by centrifugation at 15,000 rpm for 10 min. Isolated cilia were imaged using differential interference contrast microscopy with a 100× objective lens, and their lengths were measured using the line tool of ImageJ. Only cilia that were in focus along their entire length were included in the analysis. In ciliary regeneration experiments, deciliated cells were washed twice with SPP medium to remove residual dibucaine and were then resuspended in SPP medium to the original culture volume. Cells were subsequently incubated without shaking at 30°C. Additionally, for SDS-PAGE and Western blotting, collected cilia were washed twice in Tris-Mg buffer (30 mM Tris-HCl [pH 8.3] and 2 mM MgSO_4_) at 15,000 rpm for 10 min.

**Ciliary fractionation assay**

The ciliary fractionation assay was performed according to the previously described procedure (Ueno et al., 2003; Gibbons, 1963), with some modifications. Isolated cilia were treated with a solution containing 1% Triton X-100, 30 mM Tris-HCl (pH 8.3), and 2 mM MgSO_4_ on ice for 10 min and centrifuged at 15,000 rpm for 20 min. The supernatant was collected as the membrane–matrix fraction. The pellets were extracted with a solution containing 1 mM Tris-HCl (pH 8.3) and 0.1 mM EDTA on ice for 3 h and centrifuged at 15,000 rpm for 20 min. The resulting supernatant was collected as the crude-dynein fraction. The pellets were successively treated on ice for 1 h with buffers (30 mM Tris-HCl [pH 8.3] and 2 mM MgSO_4_) containing 200, 400, 500, and 600 mM KCl, followed by 600 mM KI, respectively. In each extraction step, the supernatant obtained by centrifugation at 15,000 rpm for 20 min was collected as the outer-doublet microtubule fraction. The pellets left at the end were the insoluble fraction.

**Western blotting**

Cells were cultured in SPP medium to the log phase and deciliated by dibucaine treatment, followed by centrifugation to separate deciliated cell bodies and cilia. EzApply (ATTO) was added to each fraction, and samples were boiled at 95°C for 5 min. Proteins were separated by SDS–PAGE using a 7.5% or 10% polyacrylamide gel and transferred to a ClearTrans® nitrocellulose membrane (pore size of 0.45 µm; FUJIFILM Wako Pure Chemical Corporation). After transfer, the membrane was blocked for 30 min with 1% skim milk in TTBS buffer (137 mM NaCl, 2.7 mM KCl, 25 mM Tris, and 0.1% Tween 20) (TTBS+S). The membrane was incubated overnight at 4°C in TTBS+S with the anti-mouse GFP antibody (Roche) diluted to 0.2 µg/mL. After three washes with TTBS buffer, the membrane was incubated at room temperature for 2 h in TTBS+S with the HRP-conjugated anti-mouse IgG secondary antibody (Promega) diluted 1:5,000. The membrane was washed three times with TTBS buffer. EzWestLumi One (ATTO) was applied to the membrane and evenly distributed, and chemiluminescent signals were detected using an AE-9300H Ez-Capture MG image analyzer (ATTO).

**Supplementary discussion**

In the present study, we proposed that tArp was conserved in species belonging to SAR. However, several organisms belonging to SAR were identified in which tArp could not be detected in their genomes (Table S3). Therefore, we investigated the possibility that these organisms secondarily lost tArp by focusing on the structure and function of their cilia.

Species lacking tArp were frequently observed in Apicomplexa, one of the groups constituting Alveolata. tArp was discovered in *Toxoplasma gondii* and its close relatives, but not in *Cryptosporidium* or Hematozoans, a monophyletic group within Apicomplexa including *Plasmodium*, *Theileria*, and *Babesia*. Ciliates swim using cilia that cover their cell surface, whereas dinoflagellates and the motile cells of chrompodellids move with two flagella (Adl et al., 2019). In contrast, Apicomplexa lack flagella during most stages of their life cycle, except on male gametes (Francia et al., 2015). In the male gametes of *Toxoplasma* and *Hammondia*, in which tArp was identified, flagella are formed (Francia et al., 2015; Dubey and Sreekumar, 2003). Furthermore, flagella-like structures have been identified in the male gamonts of *Neospora* (Kul et al., 2015) that possess tArp. However, species lacking flagella in their male gametes also exist in Apicomplexa, such as *Cryptosporidium*, *Babesia*, and *Theileria*, but have bundles of microtubules considered to be vestigial axonemes (Morrissette et al., 2023; Tandel et al., 2019). tArp has not been identified in these families. Therefore, the presence of flagella appears to correlate with that of tArp. The only exception was *Plasmodium*, in which flagella formed in the male gametes (Francia et al., 2015); however, tArp was not detected in the present study. Intraflagellar transport (IFT) is critically important for the formation and maintenance of cilia and flagella (Satir, 2017; Taschner et al., 2012), and is highly conserved in many eukaryotes. In *Toxoplasma*, genes encoding kinesins and IFT complex proteins involved in IFT are almost completely conserved. In contrast, these genes are not conserved in *Plasmodium* (Wickstead and Gull, 2007; Briggs et al., 2004), and flagellar assembly appears to occur within the cytoplasm via a different mechanism from IFT in this organism (Sinden et al., 2010; Briggs et al., 2004). In addition, *Plasmodium* flagella lack the major components typically found in flagella. Proteomic analyses of the reproductive cells of *Plasmodium* have identified heavy, light, and intermediate chains of three types of outer arm dyneins, whereas few proteins consisting of inner arm dyneins and the radial spoke have been detected (Talman et al., 2014; Sinden et al., 2010). In comparative genomic analyses of dynein components, the 1α heavy chain of the double-head inner arm dynein was not identified in *Plasmodium*, whereas only the 1β heavy chain was detected, and none of the analyzed single-head inner arm dyneins were found (Wickstead and Gull, 2007). Moreover, IC138 and IC140, which are well-conserved components of the double-head inner arm dynein, were not identified (Wickstead and Gull, 2007). Consistent with these biochemical data, electron microscopy of flagella revealed that electron density corresponding to inner arm dynein was lower than that of outer arm dynein (Talman et al., 2014; Sinden et al., 2010). Therefore, *Plasmodium* may have undergone distinct evolutionary pathways for flagellar formation and the structural patterns of inner arm dynein from those of other Apicomplexa. It is also possible that tArp may have been lost in this family.

Several species lacking tArp were also found in Rhizaria and Stramenopiles. In Rhizaria, tArp was discovered in Cercozoa, Endomyxa, and Aquavolonida, but not in Retaria. Genomic information for Retarian species is still limited compared to other groups, thus, the absence of tArp in this group cannot be concluded. Further genomic analyses of Retaria are expected to clarify whether tArp is present. In Stramenopiles, tArp was discovered in species belonging to Bigyra, Oomycete, and Ochrophyta, but not in diatoms. Stramenopiles typically possess two flagella, termed the anterior and posterior flagella, in their vegetative cells or in their zoospores and gametes (Safonov, 2024; Adl et al., 2019). However, diatoms lacking tArp are an exception. Diatoms are broadly classified into centric diatoms and pennate diatoms; neither possess flagella in their vegetative stages, with flagella only appearing in the male gametes of centric diatoms (Nanjappa et al., 2017). The flagella of these centric diatoms are specialized, possessing only a single anterior flagellum, and their axoneme adopts a 9+0 structure, which differs from the typical 9+2 structure (Safonov, 2024; Idei et al., 2013). Furthermore, a comparative genomic analysis of dynein components identified all components of the outer arm dynein from centric diatoms, whereas components of the inner arm dynein were not identified (Wickstead and Gull, 2007). Therefore, centric diatom flagella may lack not only the central pair of microtubules, but also the inner arm dynein. Moreover, genome surveys of IFT components indicated that many IFT-related proteins were missing in centric diatoms (Ban et al., 2023; Nanjappa et al., 2017). The central diatom *Thalassiosira pseudonana* and the pennate diatom *Phaeodactylum tricornutum* used in this phylogenetic analysis have had their genomes extensively examined (Giguere et al., 2022; Filloramo et al., 2021; Bowler et al., 2008; Armbrust et al., 2004). In addition, in the EukProt database, from which the sequences were obtained, the sequences of both *T. pseudonana* and *P. tricornutum* are included in ‘The Comparative Set’, which comprises sequences from model organisms and phylogenetically important taxa with sufficiently high BUSCO scores (Richter et al., 2022; <https://evocellbio.com/eukprot/>). Therefore, tArp is not considered to be conserved in these species. tArp was discovered in Bolidphyceae, the closest sister group to diatoms. Bolidophyceans are reported to alternate between a non-motile stage, enclosed by siliceous plates, and a motile stage that swims using two flagella (Safonov, 2024; Ichinomiya, 2016). Moreover, Bolidophyceans retain well-conserved proteins related to IFT (Ban et al., 2023). In light of previous reports and our results, diatoms may have lost tArp around the time of the loss of axonemal components and IFT-related proteins during the stage of their common ancestor or early divergence.

**Supplementary References**

Adl SM, Bass D, Lane CE, Lukeš J, Schoch CL, Smirnov A, Agatha S, Berney C, Brown MW, Burki F, Cárdenas P, Čepička I, Chistyakova L, Del Campo J, Dunthorn M, Edvardsen B, Eglit Y, Guillou L, Hampl V, Heiss AA, Hoppenrath M, James TY, Karnkowska A, Karpov S, Kim E, Kolisko M, Kudryavtsev A, Lahr DJG, Lara E, Le Gall L, Lynn DH, Mann DG, Massana R, Mitchell EAD, Morrow C, Park JS, Pawlowski JW, Powell MJ, Richter DJ, Rueckert S, Shadwick L, Shimano S, Spiegel FW, Torruella G, Youssef N, Zlatogursky V, Zhang Q. (2019). Revisions to the classification, nomenclature, and diversity of eukaryotes. J Eukaryot Microbiol. 66(1):4-119.

Angus SP, Edelmann RE, Pennock DG. (2001). Targeted gene knockout of inner arm 1 in *Tetrahymena thermophila*. Eur J Cell Biol. 80(7):486-97.

Armbrust EV, Berges JA, Bowler C, Green BR, Martinez D, Putnam NH, Zhou S, Allen AE, Apt KE, Bechner M, Brzezinski MA, Chaal BK, Chiovitti A, Davis AK, Demarest MS, Detter JC, Glavina T, Goodstein D, Hadi MZ, Hellsten U, Hildebrand M, Jenkins BD, Jurka J, Kapitonov VV, Kröger N, Lau WW, Lane TW, Larimer FW, Lippmeier JC, Lucas S, Medina M, Montsant A, Obornik M, Parker MS, Palenik B, Pazour GJ, Richardson PM, Rynearson TA, Saito MA, Schwartz DC, Thamatrakoln K, Valentin K, Vardi A, Wilkerson FP, Rokhsar DS.(2004). The genome of the diatom *Thalassiosira pseudonana*: ecology, evolution, and metabolism. Science. 306(5693):79-86.

Attwell GJ, Bricker CS, Schwandt A, Gorovsky MA, Pennock DG. (1992). A temperature-sensitive mutation affecting synthesis of outer arm dyneins in *Tetrahymena thermophila*. J Protozool. 39(2):261-6.

Ban H, Sato S, Yoshikawa S, Yamada K, Nakamura Y, Ichinomiya M, Sato N, Blanc-Mathieu R, Endo H, Kuwata A, Ogata H. (2023). Genome analysis of Parmales, the sister group of diatoms, reveals the evolutionary specialization of diatoms from phago-mixotrophs to photoautotrophs. Commun Biol. 6(1):697.

Bowler C, Allen AE, Badger JH, Grimwood J, Jabbari K, Kuo A, Maheswari U, Martens C, Maumus F, Otillar RP, Rayko E, Salamov A, Vandepoele K, Beszteri B, Gruber A, Heijde M, Katinka M, Mock T, Valentin K, Verret F, Berges JA, Brownlee C, Cadoret JP, Chiovitti A, Choi CJ, Coesel S, De Martino A, Detter JC, Durkin C, Falciatore A, Fournet J, Haruta M, Huysman MJ, Jenkins BD, Jiroutova K, Jorgensen RE, Joubert Y, Kaplan A, Kröger N, Kroth PG, La Roche J, Lindquist E, Lommer M, Martin-Jézéquel V, Lopez PJ, Lucas S, Mangogna M, McGinnis K, Medlin LK, Montsant A, Oudot-Le Secq MP, Napoli C, Obornik M, Parker MS, Petit JL, Porcel BM, Poulsen N, Robison M, Rychlewski L, Rynearson TA, Schmutz J, Shapiro H, Siaut M, Stanley M, Sussman MR, Taylor AR, Vardi A, von Dassow P, Vyverman W, Willis A, Wyrwicz LS, Rokhsar DS, Weissenbach J, Armbrust EV, Green BR, Van de Peer Y, Grigoriev IV. (2008). The *Phaeodactylum* genome reveals the evolutionary history of diatom genomes. Nature. 456(7219):239-44.

Briggs LJ, Davidge JA, Wickstead B, Ginger ML, Gull K. (2004). More than one way to build a flagellum: comparative genomics of parasitic protozoa. Curr Biol. 14(15):R611-2.

Dubey JP, Sreekumar C. (2003). Redescription of *Hammondia hammondi* and its differentiation from *Toxoplasma gondii*. Int J Parasitol. 33(13):1437-53.

Filloramo GV, Curtis BA, Blanche E, Archibald JM. (2021). Re-examination of two diatom reference genomes using long-read sequencing. BMC Genomics. 22(1):379.

Francia, M.E., Dubremetz, JF. & Morrissette, N.S. (2015). Basal body structure and composition in the apicomplexans *Toxoplasma* and *Plasmodium*. Cilia. 5: 3.

Giguere DJ, Bahcheli AT, Slattery SS, Patel RR, Browne TS, Flatley M, Karas BJ, Edgell DR, Gloor GB. (2022). Telomere-to-telomere genome assembly of *Phaeodactylum tricornutum*. PeerJ. 10:e13607.

Gibbons IR. (1963). Studies on the protein components of cilia from *Tetrahymena pyriformis*. Proc Natl Acad Sci U S A. 50(5):1002-10.

Ichinomiya M, Dos Santos AL, Gourvil P, Yoshikawa S, Kamiya M, Ohki K, Audic S, de Vargas C, Noël MH, Vaulot D, Kuwata A. (2016). Diversity and oceanic distribution of the Parmales (Bolidophyceae), a picoplanktonic group closely related to diatoms. ISME J. 10(10):2419-34.

Idei M, Osada K, Sato S, Nakayama T, Nagumo T, Mann DG. (2013). Sperm ultrastructure in the diatoms *Melosira* and *Thalassiosira* and the significance of the 9 + 0 configuration. Protoplasma. 250(4):833-50.

Kataoka K, Schoeberl UE, Mochizuki K. (2010). Modules for C-terminal epitope tagging of *Tetrahymena* genes. J Microbiol Method 82:342-6.

Kul O, Atmaca HT, Anteplioglu T, Ocal N, Canpolat S. (2015). *Neospora caninum*: the First Demonstration of the Enteroepithelial Stages in the Intestines of a Naturally Infected Dog. J Comp Pathol. 153(1):9-13.

Ludmann SA, Schwandt A, Kong X, Bricker CS, Pennock DG. (1993). Biochemical analysis of a mutant *Tetrahymena* lacking outer dynein arms. J Eukaryot Microbiol. 40(5):650-60.

Morrissette N, Abbaali I, Ramakrishnan C, Hehl AB. (2023). The Tubulin Superfamily in Apicomplexan Parasites. Microorganisms. 11(3):706.

Nanjappa D, Sanges R, Ferrante MI, Zingone A. (2017). Diatom flagellar genes and their expression during sexual reproduction in *Leptocylindrus danicus.* BMC Genomics. 18(1):813.

Richter DJ, Berney C, Strassert, JFH, Poh YP, Herman EK, Muñoz-Gómez SA, Wideman JG, Burki F, de Vargas C. (2022). EukProt: A database of genome-scale predicted proteins across the diversity of eukaryotes. Peer Community Journal. 2:e56.

Safonov P. (2024). Types of flagellar apparatus of diatomists: morphological diversity and evolution. Protistology. 18(3):177–188.

Satir P. (2017). CILIA: before and after. Cilia. 6:1.

Sinden RE, Talman A, Marques SR, Wass MN, Sternberg MJ. (2010). The flagellum in malarial parasites. Curr Opin Microbiol. 13(4):491-500.

Talman AM, Prieto JH, Marques S, Ubaida-Mohien C, Lawniczak M, Wass MN, Xu T, Frank R, Ecker A, Stanway RS, Krishna S, Sternberg MJ, Christophides GK, Graham DR, Dinglasan RR, Yates JR 3rd, Sinden RE. (2014). Proteomic analysis of the *Plasmodium* male gamete reveals the key role for glycolysis in flagellar motility. Malar J. 13:315.

Tandel J, English ED, Sateriale A, Gullicksrud JA, Beiting DP, Sullivan MC, Pinkston B, Striepen B. (2019). Life cycle progression and sexual development of the apicomplexan parasite *Cryptosporidium parvum*. Nat Microbiol. 4(12):2226-2236.

Taschner M, Bhogaraju S, Lorentzen E. (2012). Architecture and function of IFT complex proteins in ciliogenesis. Differentiation. 83(2):S12-22.

Thompson GA Jr, Baugh LC, Walker LF. (1974). Nonlethal deciliation of *Tetrahymena* by a local anesthetic and its utility as a tool for studying cilia regeneration. J Cell Biol. 61(1):253-7.

Ueno H, Gonda K, Takeda T, Numata O. (2003). Identification of elongation factor-1alpha as a Ca^2+^/calmodulin-binding protein in *Tetrahymena* cilia. Cell Motil Cytoskeleton. 55(1):51-60.

Wickstead B, Gull K. (2007). Dyneins across eukaryotes: a comparative genomic analysis. Traffic. 8(12):1708-1721.

**Supplementary Figure Legends**

**Figure S1 Construction of the EGFP-tArp construct**

Using genomic DNA from the B2086 strain as a template, a region of approximately 2.4 -kb encompassing approximately 1.4-kb upstream and 1.0-kb downstream of the start codon (black triangle) of the *tARP* gene was amplified by PCR using primers 1 and 2 (Table S2). This fragment was cloned into the pT7Blue vector (Merck Millipore) to generate the pT7Blue–tARP vector. Using the pT7Blue–tARP vector as a template, an inverse PCR product was amplified with primers 5 and 6 (Table S2). Moreover, the Neo4–MTT2–EGFP was amplified using primers 3 and 4 (Table S2). These two DNA fragments were fused by an In-Fusion reaction to generate the final construct used in the experiment shown in Figure S2. The non-coding regions and exons of *tARP* are shown in white and blue, respectively. The Neo4 cassette is derived from pNeo4 kindly provided by Dr. Mochizuki and is composed of the *MTT1* promoter + neo4 + 3’BTU2 terminator (Mochizuki, 2008).

**Figure S2 Generation of the EGFP–tArp strain and analyses of its expression and cellular localization**

**(A)** Schematic of the EGFP–tArp expression construct (a). Non-coding regions and exons of the *tARP* gene are shown in white and blue, respectively. Black triangles indicate the position of the start codon of *tARP*. Transformed cells were selected, and the complete replacement of all *tARP* genes on macronuclear chromosomes with the introduced construct was confirmed by PCR. By using primers 7 and 8 (Table S2), an approximately 2.7-kb fragment was amplified from the wild-type (WT) genome, whereas an approximately 6.7-kb fragment was amplified from the isolated strain (b). **(B)** Fluorescence images of living EGFP–tArp strain cells. Cells cultured in SPP medium without CuSO_4_ showed a weak or no detectable EGFP signal, except for weak autofluorescence from food vacuoles. In some cells, faint fluorescence was observed at the oral apparatus (white arrow), at which cilia were densely packed. On the other hand, cells cultured with CuSO_4_ at a final concentration of 1.25 mM showed fluorescence throughout the cytoplasm and cilia. The dark region in the center of the cell corresponds to the location of the macronucleus. Scale bar, 20 µm. **(C)** Western blot analysis of the EGFP–tArp strain after dibucaine treatment. Cells before (lanes 1, 2) and after Cu^2+^-induced expression (lanes 3, 4) were treated with dibucaine, separated into cell body (lanes 1, 3) and ciliary fractions (lanes 2, 4), and then subjected to SDS-PAGE (CBB-stained gel, left). A gel prepared under the same conditions was transferred to a membrane and subjected to Western blotting using an anti-GFP antibody (right). Although a faint signal was detected in uninduced cells (lane 2), a clear band corresponding to the expected molecular weight of EGFP–tArp was detected in the ciliary fraction of induced cells (lane 4). The smaller bands visible in lane 3 most likely correspond to the products of EGFP-tArp degradation.

**Figure S3 Construction of the tArp knockout construct**

Using genomic DNA from the B2086 strain as a template, an approximately 1.3-kb fragment from the 5’ flanking region of the *tARP* gene and a 1.4-kb fragment from within the *tARP* gene were amplified by PCR with primer sets 9 and 10 or 11 and 12 (Table S2), respectively. These primers contain SacI, PstI, ClaI, or XhoI recognition sites at their 5’-ends, respectively. Each PCR product was cloned into a cloning vector and subsequently excised with the corresponding restriction enzymes. The fragments were then sequentially inserted into the pNeo4 plasmid to generate the final product. The non-coding regions and exons of *tARP* are shown in white and blue, respectively. Black triangles indicate the position of the start codon of *tARP*.

**Figure S4** **Construction of tArp-EGFP**

Using genomic DNA from the B2086 strain as a template, a region of approximately 2.0 kb encompassing the stop codon (white arrowhead) of the *tARP* gene was amplified by PCR using primers 17 and 18 (Table S2). This PCR product was cloned into the pT7Blue vector (Merck Millipore) to generate the pT7Blue–*tARP_*Cter vector. Using it as a template, an inverse PCR product lacking the stop codon of *tARP* was amplified with primers 21 and 22 (Table S2). The EGFP-Neo4 cassette (Kataoka et al., 2010) was amplified using primers 19 and 20 (Table S2). These two PCR products were fused by an In-Fusion reaction to generate the final construct. The non-coding regions and exons of *tARP* are shown in white and blue, respectively.

**Figure S5 Generation of strains expressing tArp-EGFP under the control of the endogenous promoter.**

**(A)** Schematic diagram of the tArp-EGFP-expression construct (a). The *tARP* gene locus on the macronuclear chromosomes of wild-type cells (B2086) was replaced via homologous recombination with a gene fragment derived from the construct shown in Figure S4. Transformed cells were selected, and strains in which all *tARP* loci had been replaced by the construct were isolated. Using primers 23 and 24, a fragment of approximately 2.3 kb was amplified by PCR from the parent genome (left), while a fragment of approximately 5.4 kb was amplified from the isolated strain (right). **(B)** A fluorescence image of a living tArp-EGFP cell. EGFP signal was observed in both somatic and oral cilia as indicated by white and magenta arrowheads, respectively. Scale bar, 20 µm. **(C)** Western blot of tArp-EGFP. Cells were deciliated with dibucaine, and cell body (lane 1) and cilia (lane 2) proteins were analyzed by SDS-PAGE. Both fractions were applied with the same ratio relative to the original cell. To the right, a corresponding Western blot with anti-GFP antibodies. A strong band corresponding to the expected molecular weight of tArp-EGFP was detected in the ciliary fraction (lane 2). **(D)** tArp-EGFP was detected in both the crude-dynein fraction and outer-doublet microtubule fraction. Left: A CBB-stained gel showing proteins separated by SDS-PAGE from samples subjected to the fractionation experiment (see below); right: a Western blot of samples treated in the same manner using a GFP antibody. Lane 1, total proteins from isolated cilia; lane 2, the membrane-matrix fraction; lane 3, the crude dynein fraction; lanes 4–8, fractions successively extracted from outer-doublet microtubules using solutions containing 200, 400, 500, and 600 mM KCl, and 600 mM KI, respectively; lane 9, the insoluble pellet remaining after extraction with 600 mM KI.

**Figure S6 Suppressing tArp expression reduced the swimming velocity of cells**

**(A)** Representative swimming trajectories (1 s) of WT (B2086) and EGFP–tArp cells in the absence or presence of Cu^2+^ (final concentration, 1.25 mM) at 30°C. Trajectories were visualized by overlaying sequential images of swimming cells (1 s). Scale bar, 0.2 mm. **(B)** Quantification of the swimming velocity of cells. In the absence of Cu^2+^, swimming velocity was approximately 30% slower in EGFP–tArp cells than in WT cells. *P* < 0.001, the Steel–Dwass test, *N* = 150 cells. ns, not significant

**Figure S7 Ciliary beat frequency and effective stroke angle in *tARP* KO cells**

**(A)** The ciliary beat frequencies of WT, *tARP* KO, *DYH6* KO, and *OAD* mutant cells were measured using a high-speed camera at 1,000 frames per second (fps). *P* < 0.001, the Steel-Dwass test, *N* = 120 cilia (20 cells). ns, not significant. **(B)** Effective stroke angles were identified based on ciliary motion recorded with a high-speed camera at 2,000 fps. *P* < 0.001, the Steel-Dwass test, *N* = 120 cilia (20 cells). ns, not significant. Axonemal dyneins play a central role in ciliary motility and are generally classified into outer and inner arm dyneins. Outer arm dyneins generally contribute to ciliary beat frequency, whereas inner arm dyneins are responsible for forming the ciliary wave pattern. *DYH6* KO6 cells lack the α heavy chain of the two-headed inner arm (f/I1) dynein (Angus et al., 2001). The *OAD1 C11* mutant lacks outer arm dyneins and becomes immotile at a restrictive temperature of 38°C, whereas even at a permissive temperature, the partial loss of outer arm dyneins results in a slower swimming velocity than that by WT cells (Ludmann et al., 1993; Attwell et al., 1992). In *tARP* KO cells, ciliary beat frequency and the effective stroke angle were both reduced. All cells were cultured at 30°C.

**Figure S8 The loss of tArp does not significantly affect ciliary distribution, length, or regeneration**

**(A)** Immunofluorescence staining of α-tubulin. Representative images of cells are shown. TAT1 and goat anti-mouse IgG (H+L) conjugated with Alexa Fluor™ Plus 488 were used as the primary and secondary antibodies, respectively. Scale bar, 10 µm. **(B)** Ciliary length. Cilia removed from the cell body by the dibucaine treatment were collected by centrifugation. Ciliary length was measured under a microscope. The Steel-Dwass test, *N* = 150 cilia. ns, not significant. **(C)** Ciliary regeneration assay. *T. thermophila* cells regenerate cilia after deciliation. The swimming trajectories of deciliated cells were traced over time following dibucaine-induced deciliation. Immediately after deciliation, cells lost motility due to the absence of cilia. Motility gradually recovered over time. WT and *tARP* KO cells both recovered motility within approximately 3 h. Scale bar, 200 µm. All cells used in experiments were cultured at 30°C.

**Movie S1 High-speed video recording of ciliary motion**

Cell movement was recorded at 2,000 fps using a high-speed camera. All cells were cultured at 30°C. During the effective stroke, *tARP* KO and *DYH6* KO6 cells both exhibited a reduced stroke angle from that by WT cells (see Fig. S3). In WT cells, cilia during the recovery stroke moved along the cell surface, propagating the bend generated at the base horizontally to restore the cilium to its original position. In contrast, the cilia of *DYH6* KO6 cells did not move along the cell surface during the recovery stroke, but instead propagated the bend vertically. Abnormal waveforms during the recovery stroke were not observed in *tARP* KO cells.

**Supplemental Data S1**

Maximum-likelihood phylogenetic tree of actin and Arps used in this study, as shown in Figure 1. The tree is provided in the Newick format, and OTU labels are shown in the format “organism name_accession number”.
